# Glutathione peroxidase 4‐dependent glutathione high‐consumption drives acquired platinum chemoresistance in lung cancer‐derived brain metastasis

**DOI:** 10.1002/ctm2.517

**Published:** 2021-09-26

**Authors:** Wenwen Liu, Yang Zhou, Wenzhe Duan, Jing Song, Song Wei, Shengkai Xia, Yingyan Wang, Xiaohui Du, Encheng Li, Caixia Ren, Wei Wang, Qimin Zhan, Qi Wang

**Affiliations:** ^1^ Cancer Translational Medicine Research Center, The Second Hospital Dalian Medical University Dalian China; ^2^ Liaoning Clinical Research Center for Lung Cancer, The Second Hospital Dalian Medical University Dalian China; ^3^ Department of Respiratory Medicine, The Second Hospital Dalian Medical University Dalian China; ^4^ Laboratory Center for Diagnostics Dalian Medical University Dalian China; ^5^ Institute of Microelectronics Peking University Beijing China; ^6^ Key Laboratory of Carcinogenesis and Translational Research (Ministry of Education/Beijing), Laboratory of Molecular Oncology Peking University Cancer Hospital & Institute Beijing China

**Keywords:** brain metastasis, chemotherapeutic resistance, ferroptosis, glutathione metabolism, GPX4 inhibitor, lung cancer

## Abstract

**Background:**

Platinum‐based chemotherapy is effective in inducing shrinkage of primary lung cancer lesions; however, it shows finite therapeutic efficacy in patients suffering from brain metastasis (BM). The intrinsic changes of BM cells, which contribute to the poor results remain unknown.

**Methods:**

Platinum drug‐sensitivity was assessed by utilizing a preclinical BM model of PC9 lung adenocarcinoma cells in vitro and in vivo. High consumption of glutathione (GSH) and two associated upregulated proteins (GPX4 and GSTM1) in BM were identified by integrated metabolomics and proteomics in cell lines and verified by clinical serum sample. Gain‐of‐function and rescue experiments were implemented to reveal the impact and mechanism of GPX4 and GSTM1 on the chemosensitivity in BM. The interaction between GPX4 and GSTM1 was examined by immunoblotting and immunoprecipitation. The mechanism of upregulation of GPX4 was further uncovered by luciferase reporter assay, immunoprecipitation, and electrophoretic mobility shift assay.

**Results:**

The derivative brain metastatic subpopulations (PC9‐BrMs) of parental cells PC9 developed obvious resistance to platinum. Radically altered profiles of BM metabolism and protein expression compared with primary lung cancer cells were described and GPX4 and GSTM1 were identified as being responsible for the high consumption of GSH, leading to decreased chemosensitivity by negatively regulating ferroptosis. Besides, GSTM1 was found regulated by GPX4, which was transcriptionally activated by the Wnt/NR2F2 signaling axis in BM.

**Conclusions:**

Collectively, our findings demonstrated that Wnt/NR2F2/GPX4 promoted acquired chemoresistance by suppressing ferroptosis with high consumption of GSH. GPX4 inhibitor was found to augment the anticancer effect of platinum drugs in lung cancer BM, providing novel strategies for lung cancer patients with BM.

## Graphical abstract

Wnt/NR2F2/GPX4 promoted acquired chemo‐resistance by suppressing ferroptosis with high consumption of GSH and GPX4 inhibitor was found to enhance the anticancer effect of platinum drugs in lung cancer BM, providing novel strategies for lung cancer patients with BM

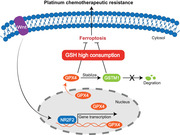

## 1 BACKGROUND

Brain metastasis (BM) is a predominant cause of death in patients suffering from nonsmall cell lung cancer (NSCLC). Patients with BM have a poor prognosis owing to the limited treatment options (median survival: 4‐6 months).^1^, ^2^ Currently available therapeutic modalities involve a combo of surgery, radiotherapy, platinum‐based chemotherapy, molecular targeted therapy, or antiangiogenesis therapy (if indicated); the outcomes are usually poor yet.^3^

Platinum‐based chemotherapy is an essential and effective treatment for patients with NSCLC;^4^ however, the therapeutic outcome in BM populations is pretty low.^5^, ^6^, ^7^, ^8^ In‐depth characterization of the mechanisms of chemotherapeutic resistance may provide insights for developing novel therapeutic strategies for patients with BM. However, the intrinsic and predisposing features of metastatic tumor cells that contribute to the unfavorable response to chemotherapy are not well characterized. Studies have investigated the internal changes in brain metastatic cells at the genetic level.^9^ However, metabolomics and proteomics are potent and prospective supporting technologies that can provide insights into the disease at a more phenomenological level.^10^, ^11^

Ferroptosis is a recently acknowledged model of nonapoptotic regulated cell death that is characterized by the production of iron‐dependent reactive oxygen species (ROS) and lipid peroxidation.^12^, ^13^, ^14^ An increasing body of evidence suggests an extremely complex relationship between ferroptosis and cancer.^15^, ^16^, ^17^, ^18^ Moreover, recent studies have revealed the involvement of ferroptosis in the process of chemoresistance. Ferroptosis has been proposed as a novel pharmacological mechanism of antitumor drugs including cisplatin (a classical platinum drug).^19^, ^20^ Glutathione peroxidase 4 (GPX4) has been shown to be a vital negative regulator of ferroptosis through its phospholipid peroxidase activity in glutathione (GSH) metabolism; GSH is an essential substrate for GPX4 to suppress lipid peroxidation.^21^, ^22^ It is suggested that tumor cells that persistently exhibit multidrugs tolerance are sensitive to GPX4 inhibition by inducing ferroptosis;^23^ however, the specific effects of GPX4 in the chemotherapy activity, especially in secondary BM of lung cancer remain obscure.

In this study, we conducted concomitant metabolomics and proteomics study in lung cancer cell line and its derived brain metastatic subpopulations to characterize the metabolic and protein expression profile in BM. We identified that the metastatic cells reprogram the cellular metabolism into a GSH‐high‐consumption state caused by the overexpression of two proteins that take part in the GSH metabolism pathway [GPX4 and its regulatory target protein glutathione S‐transferase M1 (GSTM1)]. This phenomenon contributes to the acquisition of chemotherapeutic resistance through inhibition of ferroptosis. Inhibition of GPX4 expression and its activity in vitro and in vivo was found to enhance the anticancer effect of platinum drugs in brain metastatic cells. Further, we found that the activation of Wnt/NR2F2 signaling is responsible for the transcriptional upregulation of GPX4 in BM cells.

## 2 METHODS

The detailed methods for Western blot analysis, RNAi and plasmid design and transfection, enzyme‐linked immunosorbent assay (ELISA), quantitative‐polymerase chain reaction (q‐PCR), confocal immunofluorescence, and immunohistochemistry (IHC) staining are available in the Supplemental Materials.

### 2.1 Cell culture and drugs

The human lung cancer cell line PC9 was obtained from the Chinese Academy of Medical Sciences (Beijing, China). The BM derivative cells (PC9‐BrM1, PC9‐BrM2, and PC9‐BrM3) were derived from parental cell line PC9 by implanting tumor cells in immunodeficient mice via left ventricular injection; the metastatic cells were extracted from the harvested brain metastases as described in previous work.^9^ PC9‐BrM3 was created with two more rounds of injection‐extraction‐expansion cycling; it has been shown to exhibit enriched brain metastasis activity.^24^, ^25^ These cells were cultured in Roswell Park Memorial Institute medium‐1640 (RPMI1640) with the supplementation of 10% fetal bovine serum (FBS), 100 U/mL penicillin and streptomycin (all those agents were obtained from Gibco, Invitrogen, Inc, USA) and a humidified environment with 5% CO2 at 37°C was used to maintain.

Cisplatin (MB1055), Carboplatin (MB1297), Ferrostatin‐1 (Fer‐1, MB4718), (1S, 3R)‐RSL3 (RSL3, MB4723), TGF‐β inhibitor SB431542 (MB5459), Wnt/β‐catenin inhibitor I WR‐1‐endo (MB4050), p38 MAPK inhibitor SB203580 (MB5062), and ERK inhibitor SCH772984 (MB3432) were purchased from Meilunbio (China). Wnt agonist 1 (B6059), Cycloheximide (CHX, A8244) was obtained from ApexBio (USA).

### 2.2 Cell viability and colony formation assay

Cell viability was quantified using the Cell Counting Kit‐8 (K1018, ApexBio, USA) as per the instructions of the manufacturer. For colony formation, 10^3^ cells were harvest and put into 6‐well plates and allow to proliferate for 10 days. The obtained colonies were fixed using paraformaldehyde (4%) for 10 minutes and stained with crystal violet (1%) for 20 minutes.

### 2.3 Animal studies

The licensing committee of Dalian Medical University authorized the animal studies. Vital River Laboratory Animal Technology Company (Beijing, China) provided the immunodeficient mice (female, BALB‐c‐nu, 4‐6 weeks) needed for the studies.

### 2.4 Subcutaneous xenograft studies

Five million parental cells PC9 or highly brain metastasis subpopulation PC9‐BrM3 in 100 μL phosphate buffered saline (PBS) were injected subcutaneously (s.c.) to the dorsal left flank of nude mice, respectively (*n* = 10 per group). Each group was randomly divided into a control group (*n* = 5) and a dosing group (*n* = 5). The therapy is launched when the tumor volume amounts to 100‐150 mm^3^. Mice in the control group were treated with DMSO while those in the dosing group were intraperitoneally (i.p.) injected with 5 mg/kg cisplatin once every 5 days.

**HIGHLIGHTS**

1. Lung cancer brain metastatic cells obtain a significant resistance to platinum drugs.
2. A notable GSH high‐consumption state exists in lung cancer BM.
3. GPX4 and GSTM1 contribute to the consumption of GSH and mediate the platinum resistance by suppressing ferroptosis.
4. Wnt/NR2F2 signaling is responsible for transcriptional upregulation of GPX4.

In another experiment, 5 million highly brain metastasis cells PC9‐BrM3 with negative control shRNA (shNC) or GPX4 knockdown (shGPX4) in 100 μL PBS were injected subcutaneously to the dorsal left flank of nude mice, respectively. The therapy is launched when the tumor volume amounts to 100‐150 mm^3^. Mice planted with BrM3‐shNC cells were treated with DMSO (*n* = 5, once every 5 days, i.p.) or 5 mg/kg cisplatin (*n* = 5, once every 5 days, i.p.) with or without 100 mg/kg RSL3 (*n* = 5 per group, twice a week, s.c. at tumor site), while another five mice were administered RSL3 alone. At the same time, mice planted with BrM3‐shGPX4 cells were treated with DMSO (*n* = 5, once every 5 days, i.p.) or 5 mg/kg cisplatin (*n* = 5, once every 5 days, i.p.) with or without 0.2 mg/kg ferrostatin‐1 (*n* = 5 per group, daily, i.p.), while another five mice were administered ferrostatin‐1 alone. Tumor volume was determined every 3 days according to the following formula: volume (*V*) = 1/2 × *length* × *width* ^2^. Mice were sacrificed and masses were removed on the 21st day of treatment.

### 2.5 Intracardiac BM models

One million PC9‐BrM3 cells with GPX4 knockdown (shGPX4) in 100 μL PBS were administered into the left ventricle of each mouse after anesthesia. Weekly analysis of brain colonization by bioluminescence imaging (BLI) was performed in vivo. Briefly, images were obtained within 15 minutes after intraperitoneal administration of D‐luciferin (150 mg/kg body weight; Promega, USA) into anesthetized mice using an IVIS Spectrum Xenogen machine (PerkinElmer, USA). The Living Image software (version 2.50) was utilized for analyzing the images. Drug treatment over a 21‐day period was initiated after confirmation of colonization; mice were treated with DMSO (*n* = 3, once every 5 days, i.p.) or 5 mg/kg cisplatin (*n* = 3, once every 5 days, i.p.) with or without 0.2 mg/kg ferrostatin‐1 (*n* = 3 per group, daily, i.p.), while another three mice were administered ferrostatin‐1 alone.

### 2.6 Clinical samples

The Ethics Review Committee of the Second Hospital of Dalian Medical University approved this study. Serum samples were obtained from lung cancer patients with brain metastasis (LCBM, *n* = 108) and patients with primary lung cancer with no lymph node or distant organ metastasis (PLC, *n* = 40) following a standard operating protocol. Briefly, 2 mL peripheral blood in a serum separator tube (SST) was allowed to stand still for 30 minutes (for clotting), followed by centrifugation (1000 g/min) for 15 minutes at room temperature. Immediately after centrifugation, serum was collected into clean polypropylene tubes and stored at –80°C. Written informative consent was received from all participating individuals.

### 2.7 Metabolic profiling analysis

The PC9 and its derivative subpopulations (PC9‐BrMs) were cultured in 10‐cm dish with 5‐6 replicates per group. After achievement of 80%‐90% confluence, cells were promptly washed thrice with ice‐cold PBS, harvested with 1 mL of methanol/water solution (4:1, v/v), and vortex‐treated. 350 μL of the supernatant solution were collected after centrifugation and lyophilized. For serum samples, 50 μL of serum was extracted with 200 μL of methanol solution containing 5 μg/mL of myristic acid‐d27 as inner standard and vortex‐processed. 180 μL of the supernatant solution was collected after centrifugation and lyophilized. Before GC‐MS analysis, the lyophilized samples were treated with oximation and silylation reactions to improve the volatility of metabolites. Briefly, cell and serum lyophilized samples were subjected to sonication after addition of 30 μL and 50 μL of a methoxamine solution (20 mg/mL in pyridine), respectively, and incubated at 37°C for 1.5 hour. Subsequently, 20 and 40 μL of N‐methyl‐N‐(trimethylsilyl)‐trifluoroacetamide (MSTFA), respectively, were added and incubated at 37°C for 1 hour. After centrifugation, supernatant was obtained and used for further profiling. Quality‐control (QC) samples were produced by bringing together identical aliquots of each cell and serum sample, respectively, and were pretreated as the samples.

The metabolic profiling was performed using a GCMS‐QP2010 plus system (Shimadzu, Kyoto, Japan) combined with a DB‐5 MS fused‐silica capillary column (Agilent Technologies, Palo Alto, CA, USA), and a GCMS‐TQ8050 system (Shimadzu, Kyoto, Japan) coupled with a Rxi‐5Sil MS fused‐silica capillary column (Restek, Bellefonte, PA, USA) for cell and serum samples, respectively. One microliter of samples was injected at a 1:10 split ratio. The linear velocity of carrier gas (Helium, 99.9995 %, China) was set at 40 cm/s. The oven temperature was maintained at 70°C for 3 minutes and then ramped to 300°C with temperature increment of 5°C/min and kept for 10 minutes for cell samples; for serum samples, the oven temperature was kept at 80°C for 1 minute and raised to 210°C at increment of 30°C/min, then to 315°C at increment of 20°C/min and maintained for 5 minutes. An electron ionization source (EI, 70 eV) was employed for ionization. The detection voltage was programmed based on the outcomes of the autotuning. Data access for cell and serum samples was started at 5.6 and 2.92 minutes, respectively, with the mass scan range of 50‐600 *m*/*z* and the event time of 0.2 second. The temperature of the ion source was 230°C. The temperatures of the inlet and the transfer line were 300°C and 280°C for cell samples, respectively, and 315°C and 300°C for serum samples, respectively. The sample injection was in random order, and a QC sample was analyzed every 6 cell and 10 serum samples, respectively, to monitor the data quality.

### 2.8 Measurement of glutathione

A total of 5000 cells were seeded in 96‐well white plates and allowed to attach for 12 hours. Glutathione (GSH) level was evaluated using the GSH‐Glo assay kit (Promega, V6912) according to the manufacturer's instructions. To detect total glutathione (GSH + GSSG), reducing agent tris(2‐carboxyethyl) phosphine (TCEP) (which can reduce oxidized glutathione [GSSG]) was used at a concentration of 500 μM following the manufacturer's guidelines. Each measurement was read out by a multifunctional microplate reader (Varioskan LUX, Thermo).

### 2.9 Analysis of intracellular reactive oxygen species

Reactive Oxygen Species (ROS) assay kit (Beyotime, S0033S) was used to measure intracellular ROS level on the basis of the formation of fluorescent compound 2 #, 7 # ‐dichlorofluorescein (DCF) under the peroxide‐dependent oxidation of DCFH‐DA, according to the manufacturer's guidelines. A total of 5000 cells/well were planted in 96‐well plates and then subjected to specific drug treatments upon cell attachment. After the treatment, 200 μL DCFH‐DA (10 μM) was added to each well after removing media containing the drug and left to 20 minutes of warming incubation at 37°C. Remove the residual DCFH‐DA by washing cells with serum‐free cell culture medium. Fluorescence intensity was determined with a multifunctional microplate reader (Varioskan LUX, Thermo). Since the brain metastasis populations PC9‐BrM3 exhibited green fluorescence,^[^ ^25^ ^]^ the data were normalized to the respective unstained cells.

### 2.10 Proteomic analysis

It was performed with the support of Jingjie PTM Biolabs (Hangzhou, China) Co. Ltd. The detailed laboratory protocols for protein profiling are described in the Supplementary Materials.

### 2.11 Lipid peroxidation assay

Fluorescent Dye C11 BODIPY 581/591 (D3861, Invitrogen) was used as lipid peroxidation sensor as per the manufacturer's instructions. Briefly, 50,000 cells were seeded on 35‐mm glass bottom dishes and then subjected to the indicated treatments upon attachment. After treatments, cells were stained with 10 μM lipid peroxidation sensor and Hoechst 33342 (Meilunbio, China) for 30 minutes. The cells were then washed for three times with PBS and then imaged on an Olympus IX81 inverted microscope using a 40× objective using filters for Hoechst, FITC, and Texas Red channels. The fluorescent signal was quantified by SlideBook™ 5.0 software, which set the ratio of signals from 510 and 590 channels to quantify lipid peroxidation in cells. Tert‐butyl hydroperoxide (TBH, 200‐500 μM for 1‐3 hours) treatment was as administered a positive control.

Lipid Peroxidation Assay Kit (ab118970, Abcam) was used to evaluate the relative concentration of malondialdehyde (MDA) in cell lysates following the manufacturer's instructions. Data were normalized by corresponding protein concentration.

### 2.12 Iron measurements

Total iron (Fe^2+^ and Fe ^3+^) was determined using the colorimetric Iron Assay Kit (ab83366, Abcam) as per the manufacturer's instructions. For three biological replicate samples, 1.5 million cells were tested per triplicate. Colorimetric was read out using a multifunctional microplate reader (Varioskan LUX, Thermo). Iron measurements for tissues were standardized by cell quantities.

### 2.12 Coimmunoprecipitation

After cell lysis, the lysates were incubated with antibodies against GPX4 or GSTM1 sufficiently at 4°C for 1 hour and the immunocomplex was then precipitated with a Capturem IP & Co‐IP Kit (Takara Bio, Japan) according to the manufacturing guidelines. After being washed, the proteins were identified by Western blot analysis using anti‐GPX4 and anti‐GSTM1.

### 2.13 Dual‐luciferase reporter assays

The luciferase reporter constructs containing the human GPX4 promoter fragments were prepared using the pGL4‐basic vector (Promega) by GenePharma (China). 293T cells were transiently transfected with the desired pGL4 basic‐based construct, NR2F2 pcDNA3.1 plasmid (GenePharma, China), and Renilla luciferase reporter vector (RL‐TK) using the Lipofectamin 2000 (Invitrogen) following the protocols. The luciferase activity was assessed using the Dual‐Glo^®^ Luciferase Assay Kit (E1910, Promega) with a microplate reader (Perkin Elmer).

### 2.14 Chromatin immunoprecipitation (ChIP)

ChIP analysis was performed using the ChIP kit (p‐2002, EpQuikTM) as per the manufacturing guidelines. Chromatin extracts containing DNA fragments were immunoprecipitated with 3 μg of monoclonal anti‐NR2F2 antibody (ab211777, Abcam) or normal IgG (as a control).

The PCR primers were as follows: forward, 5′‐ATGGCAGGGGTGAGGGTA‐3′, reverse, 5′‐ATTTTTAGTAGAGACGGGGTT‐3′ (product length 228 bp).

### 2.15 Electrophoretic mobility shift assay (EMSA)

EMSA was performed using the Lightshift Chemiluminescent EMSA Kit (Thermo Scientific, #20148) according to the manufacturer's protocol. The sense probe sequences for EMSA were as follows: wild‐type probe: 5′‐ GCGGGCAGATCACCTGAGGTCAGGAGTTCGAGA‐3′; mutant probe: 5′‐GCGGGCAGATCACAGTCTTGACTTCGTTCGAGA ‐3′. After synthesizing the double‐stranded (ds) probes and labeling the end of the wild‐type probe with digoxigenin‐11‐ddUTP, nuclear protein (5 mg) was extracted using a kit (Thermo Scientific) and then applied into the incubation containing 1 mg poly (d [I‐C]), the binding buffer, labeled wild‐type probe with or without unlabeled probe for 15 minutes at room temperature. The bound DNA complexes were electrophoretically isolated by 5% nondenaturing polyacrylamide gel and transferred to a nylon membrane. The nylon films were cross‐linked and examined for chemiluminescence by CSPD. The signals were documented with the BIO‐RAD system. In supershift analyses, after the addition of the probe, the NR2F2 antibody (4 mg; Abcam) was incorporated into the nuclear extract and incubated at 4°C for 1 hour.

### 2.16 Statistical analysis

For cell metabolomics, identification and quantification of metabolic features were performed using the chromTOF 4.43 (LECO, Saint Joseph, USA) and GC−MS browser (Shimadzu, Kyoto, Japan) software, respectively. The intensity of metabolic features was normalized to the total peak areas of raw data from cell samples, and the intensity of features that belong to one metabolite were added. For serum cystine analysis, the intensity of cystine was quantified based on the characteristic ion 218 *m*/*z*, then normalized to the intensity of myristic acid‐d_27_. The partial least‐squares discriminant analysis (PLS‐DA) with unit variance (UV) scaling was conducted by SIMCA 13.0 (Umetrics, Umea, Sweden), and was verified by a permutation test with 199 cycles.

GraphPad Prism software 5.0 and SPSS 16.0 were used for statistical analysis. Quantification data are expressed as mean (±SD) values from a minimum of three individual experiments. Student's *t* test was used for differences analysis between two groups while one‐way analysis of variance (ANOVA) was used for comparison of more than two groups. The correlation between cysteine levels and GPX4 expression levels in serum samples was assessed using the Spearman's rank correlation test.

## 3 RESULTS

### 3.1 Highly brain metastatic lung cancer cells showed significant resistance to platinum‐based chemotherapy

**FIGURE 1:**
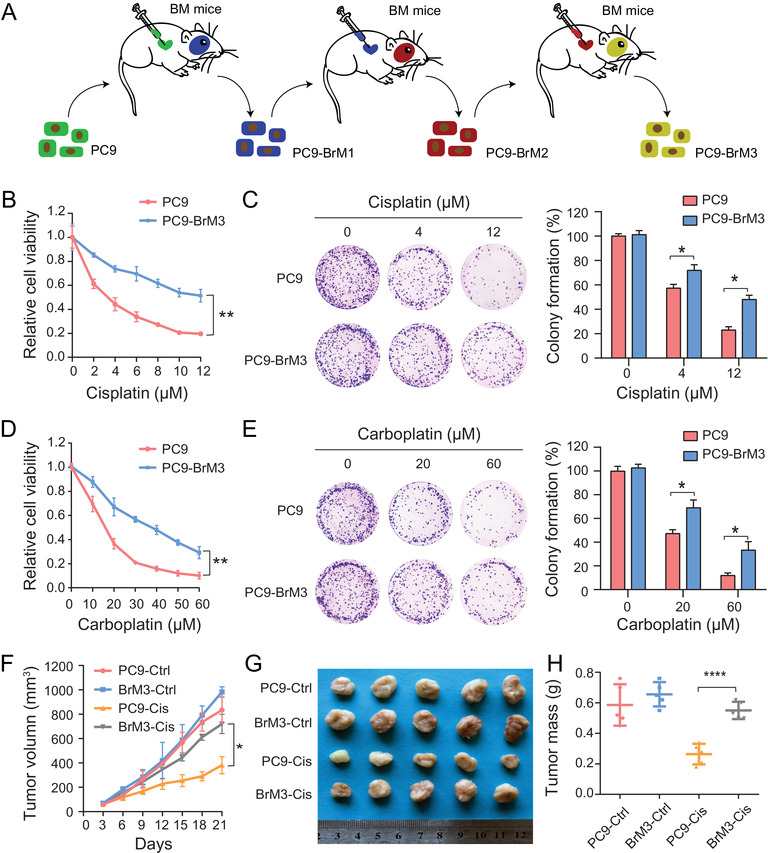
Highly brain metastatic lung cancer cells showed significant resistance to platinum‐based chemotherapeutic agents. (A) Schematic illustration of the in vivo isolation of metastatic populations (BrM1, BrM2, and BrM3) from the PC9 lung adenocarcinoma line and the subsequent metabolomics and proteomics analyses. (B, D) PC9 and PC9‐BrM3 cells were treated with different doses of cisplatin (B) or carboplatin (D) for 72 hours and CCK‐8 assays were performed to determine their viability (*n* = 3, **P < 0.01). (C, E) Survival of PC9 and PC9‐BrM3 cells after treatment with certain concentrations of cisplatin (0, 4, 12 μM) (C) or carboplatin (0, 20, 60 μM). (E) Results of clonogenic assays showing the treatment effect. Pixel density quantification of clonogenic assays is shown as histogram (*n* = 3, *P < 0.05). (F‐H) Nude mice were injected subcutaneously with the indicated PC9 or PC9‐BrM3 cells (5×10^6^ cells/mouse) and treated with cisplatin (5 mg/kg/i.p., once every 5 days) or with DMSO as control (Ctrl) (*n* = 5 mice/group). Tumor size was measured every 3 days using caliper to plot the growth curve. (E) The tumor mass was weighed at the end of the experiment (F and G). (*P < 0.05, ****P < 0.0001)

The brain metastatic subpopulations PC9‐BrM1, PC9‐BrM2, and PC9‐BrM3 derived from parental cell line PC9 were generated by intracardiac injection to immunodeficient mice and extraction of the metastatic cells from brain metastases (Figure 1A). In our previous study, PC9‐BrM3 cells were shown to exhibit high propensity for brain metastasis.^25^ These cell models are ideal models for drug susceptibility studies because of the consistent genetic homology of these cells, which excludes the effect of heterogeneity among individual patients.

Cisplatin and carboplatin‐based platinum is the basic first‐line chemotherapy drug for lung cancer brain metastases.^3^, ^7^, ^8^, ^26^ To explore the differences between parental PC9 and highly brain metastatic PC9‐BrM3, we treated these two groups of cells with platinum drugs. The results of cell viability assay and colony formation assay indicated obvious platinum drug resistance of the highly brain metastatic PC9‐BrM3 compared with the parental PC9 group, both in the cisplatin and carboplatin groups (Figure 1B‐E). Subsequently, we evaluated the response of platinum drugs in vivo. The results were consistent with those of in vitro assays; PC9‐BrM3 showed a negative response to cisplatin compared with PC9 cells in vivo (Figure 1F‐H). Collectively, the results indicated significant platinum‐based chemotherapeutic resistance of the highly brain metastatic lung cancer cells established by us; these results were consistent with the clinical status. In addition, the results highly indicated the cellular endogenous factors gave the main contributions to the poor effect of chemotherapy in BM.

### 3.2 Metabolic profiling revealed a notable GSH high‐consumption state in lung cancer BM

**FIGURE 2:**
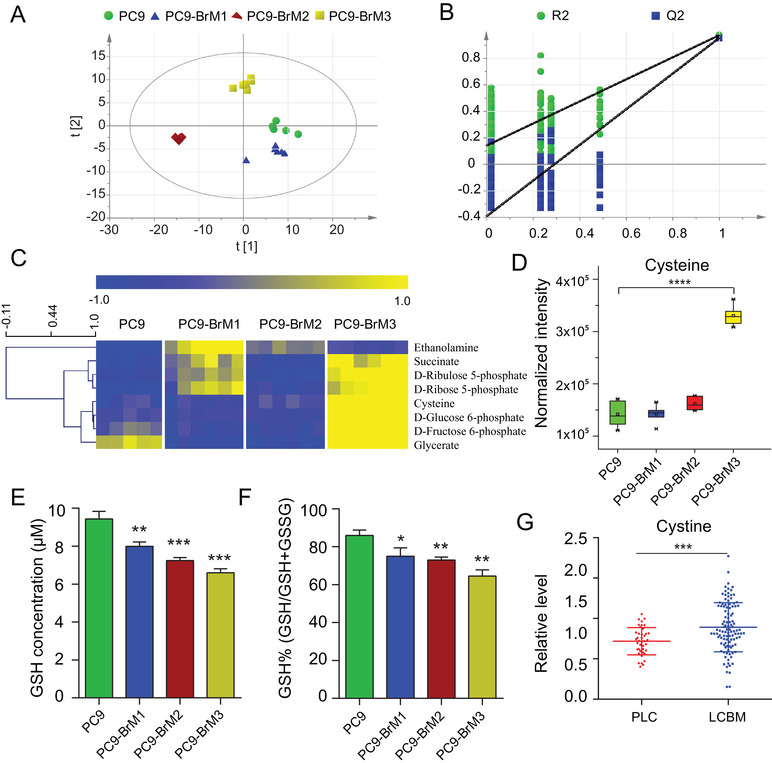
Metabolic profiling revealed a notable GSH high‐consumption state in lung cancer BM. (A) The PLS‐DA score scatter plot with UV scaling. The model parameters: R2X = 0.79, R2Y = 0.967 and Q2 = 0.952. (B) The validation plot of permutation test with 199 cycles. The R2Y and Q2 intercept values were 0.14 and –0.394, respectively. (C) Heat map of focused differential annotated metabolites in lung cancer BM. (D) Box plot of cellular cysteine levels in brain metastatic subpopulations and PC9 (****P < 0.0001). (E‐F) The levels (E) and concentrations (F) of glutathione (GSH) were assayed. The concentrations of GSH were evaluated by the ratio of GSH to the reduced plus oxidized glutathione (GSH+GSSG) levels (*n* = 3, *P < 0.05, **P < 0.01, ***P < 0.001 vs PC9 group). (G) Results of GC‐MS showing the serum cystine levels in clinical groups. Each dot corresponds to one subject (PLC, primary lung cancer, *n* = 40; LCBM, lung cancer brain metastasis *n* = 108, ***P < 0.001)

Metabolomics is a novel technique to depict the phenotypic changes in the organism and for mining the active drivers of disease evolution.^27^ We employed metabolomics to unravel the underlying cellular endogenous factors in lung cancer BM subpopulations (PC9‐BrMs) and its parental cells (PC9). Based on the gas chromatography‐mass spectrometry (GC‐MS) metabolomics described elsewhere,^28^ a typical total ion chromatogram of cell metabolic profile was displayed (Figure S1A). The relative standard deviation (RSD) distribution of QC samples illustrated a good data quality of metabolomics study (Figure S1B). For multivariate statistical analysis, we conducted partial least‐squares discriminant analysis (PLS‐DA) to uncover the global changes in metabolic profile and identified important variations in brain metastatic subpopulations (Figure 2A); the permutation test indicated the robustness of the model with no signs of overfitting (Figure 2B). The R2Y and Q2 values (indicators of the explanation and predictive ability) of the PLS‐DA model were 0.967 and 0.952, respectively. As shown in the PLS‐DA score plot (Figure 2A), the brain metastatic subpopulations (especially the PC9‐BrM2 and PC9‐BrM3) were distinctly separated from the parental cell line PC9. The features for which the variable importance in projection (VIP) values exceeded 1 contributed to the distinct separation between the disparate groups (Figure S1C). Among these, we identified and analyzed the top ten metabolic features.

Univariate analysis was performed to screen the differential features. The results showed that all ten features were differential in the PC9‐BrM3 and PC9 comparison, and most were differential in all brain subpopulations. These features were annotated by mass spectral similarity search using commercial mass spectral libraries (Mainlib, NIST, Wiley, and Fiehn) and a home‐made metabolite library, and were further validated by RT/RI using chemical standards; the details were provided in Table S1. Eight of the ten features annotated and verified by chemical standards were significantly upregulated in PC9‐BrM3, as displayed in the heat map (Figure 2C). These metabolites were mainly involved in cellular oxidative stress homeostasis associated pathways, such as pentose phosphate pathway (PPP) and glutathione metabolism. The PPP is a branch pathway of glucose metabolism; it serves an essential role in the biosynthesis of cellular reductant (nicotinamide adenine dinucleotide phosphate [NADPH]).^29^ Along with the reaction converting NADPH to NADP^+^, glutathione disulfide (GSSG) is reduced to GSH, a key antioxidant to defend against the cellular reactive oxygen species (ROS); the ROS levels in the BM subpopulations were significantly higher than those in parental PC9 cells (Figure S1D). This suggested that the brain metastatic subpopulations were in an extremely disordered oxidative stress state. Cysteine is a nonessential amino acid that is associated with oxidative stress homeostasis. It acts as the rate‐limiting precursor in the biosynthesis of GSH;^30^ in addition, it is a stable indicator of the level of GSH, since GSH can be easily oxidized leading to unstable detection results. Studies have shown that the level of cysteine is regulated by GSH via a negative feedback mechanism.^31^ We found significant increase in the level of cysteine in BM subpopulations (Figure 2C‐D), suggesting a highly GSH‐consuming phenotype of BM subpopulations. For direct confirmation of this unique status, we measured the levels of intracellular GSH and those of reduced plus oxidized glutathione (GSH+GSSG) by using the GSH kits for cell lysates. The results verified the high utilization of GSH in BM cells (Figure 2E‐F). Further, we confirmed the metabolic characteristics in clinical samples. The reduced form of cysteine was primarily intracellular, while extracellular cystine was predominantly present as an oxidized dimeric form of cysteine.^32^ Extracellular cystine is reduced to cysteine after its intracellular translocation, and the uptake of cystine is the rate‐limiting step in the synthesis of GSH.^33^, ^34^, ^35^ Therefore, clinical confirmation was performed by analyzing the cystine content in serum samples obtained from lung cancer patients with brain metastasis (LCBM, *n* = 108) and primary lung cancer patients with no lymph node or distant organ metastasis (PLC, *n* = 40) based on GC‐MS. The results showed that the cystine level in the LCBM group was consistently higher than that in the PLC group (Figure 2G). To summarize, these data demonstrated the notable GSH high‐consumption state in lung cancer BM.

### 3.3 Proteomics identified the role of GPX4 and GSTM1 in glutathione metabolism

**FIGURE 3:**
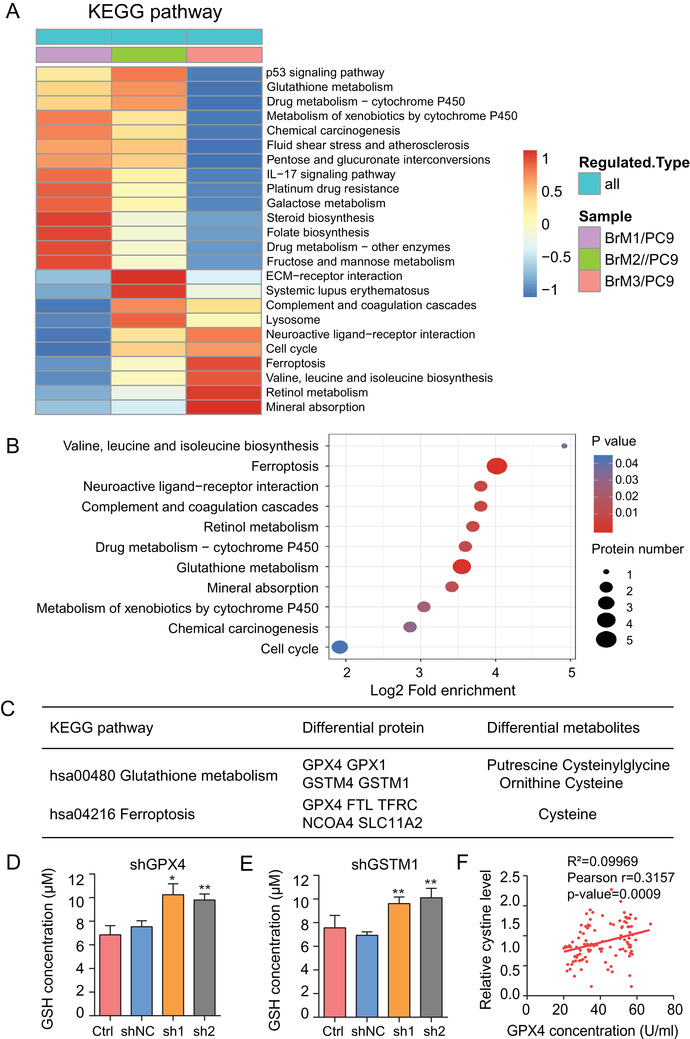
Proteomics identified the role of GPX4 and GSTM1 in glutathione metabolism. (A) Functional KEGG enrichment cluster image showing the common enriched pathways in all BM subgroups. (B) Results of KEGG enrichment analysis of differential proteins in groups (BrM3/PC9) visualized in bubble diagram (including the number of respective involved proteins and the corresponding *P*‐values). (C) The specific differential proteins and metabolites identified in groups (BrM3/PC9) involved in glutathione metabolism and ferroptosis pathways are listed. (D‐E) The levels of glutathione (GSH) were assayed (*n* = 3, *P < 0.05, **P < 0.01 versus shNC group). Ctrl, control PC9‐BrM3 cells; shNC, PC9‐BrM3 cells transfected with negative control shRNA; shGPX4, PC9‐BrM3 cells transfected with GPX4‐targeted shRNA vector; shGSTM1, PC9‐BrM3 cells transfected with GSTM1‐targeted shRNA vector; sh1, PC9‐BrM3 cells transfected with GPX4 or GSTM1‐targeted shRNA‐1; sh2, PC9‐BrM3 cells transfected with GPX4 or GSTM1‐targeted shRNA‐2. (F) Scatter diagram showing the correlation between the level of cystine and GPX4 expression in serum samples of 108 lung cancer patients with brain metastasis

Quantitative tandem mass tag (TMT)‐based proteomics can provide deep insights into the state of cellular metabolism. To explore the underlying causes of the notable GSH high‐consumption state in lung cancer BM, we performed quantitative TMT‐based proteomics along with the metabolomics study, since the cellular metabolic changes are often attributable to the action of proteins, especially the abundant metabolic enzymes.^36^ The RSD distribution of QC samples indicated good reproducibility of proteomics data (Figure S2A). We used the ratio fold (BrM/PC9) > 1.5 as the screening criteria to identify proteins in each subgroup. The number of differential proteins showed a constant decrease with enrichment of the characteristics of brain metastatic cells (Figure S2B). Differential proteins in each BM subgroup were mapped and enriched in KEGG pathways; the common enriched pathways in all BM subgroups were shown in the functional KEGG enrichment cluster image (Figure 3A). The pathway of glutathione metabolism and the closely associated ferroptosis were found enriched in BM subpopulation cells compared to the parental cells. To identify the proteins involved in these two pathways, we selected the differential proteins in the BrM3 cells, which are proven as the highly BM cells for KEGG enrichment analysis; we visualized the specific enrichment results, including the number of respective proteins involved and the corresponding *P* values, especially for glutathione metabolism and ferroptosis pathways (Figure 3B). Subsequently, we listed the differential proteins identified by proteomics and the differential metabolites identified by metabolomics involved in these two pathways of interest (Figure 3C). The results suggested that the glutathione metabolism in BM was regulated by glutathione peroxidases and glutathione S‐transferases; among these, GPX4 and GSTM1 were further verified to be stably overexpressed in BM subpopulations (PC9‐BrMs) compared to the parental PC9 cells by Western blot analysis (Figure S2C).

To confirm whether GPX4 and GSTM1 contribute to the notable GSH high‐consumption state in lung cancer BM, shRNAs targeting GPX4 or GSTM1 were transfected into PC9‐BrM3 cells while GPX4 or GSTM1 plasmids were transfected into parental PC9 cells, respectively. ELISA assays showed that the changes in GPX4 enzyme activity were consistent with the changes in protein expression under transfection whether cellular or secretory (Figure S2D‐G); subsequently, the intracellular GSH level and reduced plus oxidized glutathione (GSH+GSSG) levels were assessed. The results showed that knock‐down of GPX4 or GSTM1 reversibly increased the intracellular GSH level while overexpression of GPX4 or GSTM1 caused obvious consumption of intracellular GSH (Figures 3D‐E, S2H‐M), without any change in the other GSH regulatory proteins or enzymes (Figure S2N‐P); this illustrates that the expression of GPX4 and GSTM1 was the main reason for the consumption of GSH in lung cancer BM. Moreover, cellular GPX4 and cysteine were differentially identified both in the glutathione metabolism and ferroptosis pathways, suggesting a close connection between GPX4 and cysteine in BM cells. Therefore, we further detected the GPX4 activity with ELISA assays and investigated the correlation between GPX4 activity and cystine levels, which can sensitively reflect the level of intracellular cysteine, in the serum of lung cancer patients with BM. The results showed a significant positive correlation between cystine and GPX4 (Figure 3F); this indicated a vital role of GPX4 in the glutathione metabolism‐related ferroptosis in lung cancer BM. In general, these data suggested that the notable GSH high‐consumption state in lung cancer BM was caused by the upregulation of GPX4 and GSTM1.

### 3.4 Suppression of GPX4 and GSTM1 enhanced the platinum sensitivity of BM cells by inducing ferroptosis

**FIGURE 4:**
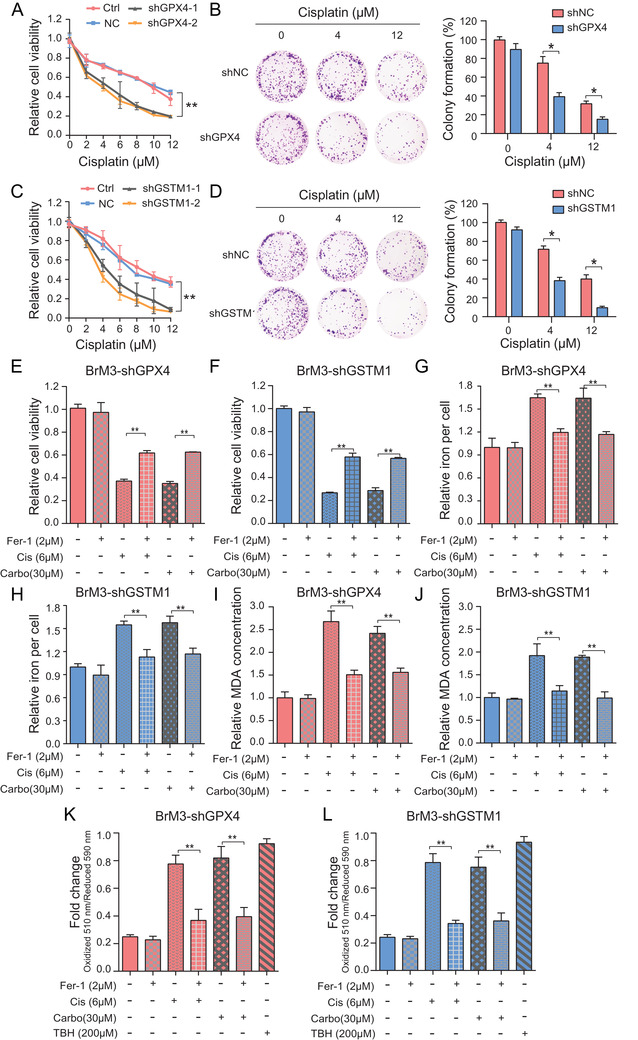
Suppression of GPX4 and GSTM1 enhanced the platinum sensitivity of BM cells by inducing ferroptosis. Indicated GPX4 (A) or GSTM1 (C) knockdown PC9‐BrM3 cells were treated with different doses of cisplatin for 72 hours and CCK‐8 assays were performed to determine their viability (*n* = 3, **P < 0.01). Results of clonogenic assays showing cell survival of the indicated GPX4 (B) or GSTM1 (D) knockdown PC9‐BrM3 cells after treatment with certain concentrations of cisplatin (0, 4, 12 μM). Pixel density quantification of clonogenic assays is shown as histogram (*n* = 3, *P < 0.05). (E‐L) Indicated GPX4 or GSTM1 knockdown PC9‐BrM3 cells were treated with cisplatin (Cis, 6 μM) or carboplatin (Carbo, 30 μM), with or without ferrostatin‐1 (Fer‐1, 2 μM) for 72 hours. The cell viability (E‐F), levels of total iron (G‐H), MDA (I‐J) and lipid peroxidation (BODIPY™ 581/591 C11 stain) (K‐L) were assayed (*n* = 3, **P < 0.01). Ctrl, control PC9‐BrM3 cells; shNC, PC9‐BrM3 cells transfected with negative control shRNA; shGPX4, PC9‐BrM3 cells transfected with GPX4‐targeted shRNA vector; shGSTM1, PC9‐BrM3 cells transfected with GSTM1‐targeted shRNA vector; TBH, tert‐butyl hydroperoxide (200 μM for 2 hours) treatment was administered as a positive control for BODIPY™ 581/591 C11 stain

GSH has been proved as an important antioxidant that protects the cells from the toxic effects of lipid peroxidation and prevents ferroptosis.^21^ Since GPX4 and GSTM1 were overexpressed in BM and active in GSH consumption, we explored the potential association between up‐regulated protein expressions and the observed acquired platinum resistance of brain metastatic cells. shRNAs targeting GPX4 or GSTM1 were transfected into PC9‐BrM3 cells and the platinum sensitivity of cells was evaluated by cell viability and clonogenicity assays. Suppression of GPX4 or GSTM1 was found to significantly enhance the response of PC9‐BrM3 to cisplatin (Figure 4A‐D) and carboplatin (Figure S3A‐D).

An increasing number of studies have uncovered that ferroptosis is a novel pharmacological mechanism and acquired drug resistance mechanism of antitumor drugs including cisplatin.^19^, ^20^, ^37^ In a recent study, cisplatin was shown to induce both ferroptosis and apoptosis in the NSCLC cell line A549.^38^ Consistently, in our study, cell death induced by both cisplatin and carboplatin was partially reversed by specific ferroptosis inhibitor Ferrostatin‐1 (Fer‐1) in parental PC9 cells (Figure S3E‐F). To determine the involvement of ferroptosis (characterized by iron accumulation and lipid peroxidation) in mediating the effect of GPX4 and GSTM1 on platinum resistance, we treated GPX4 or GSTM1 knockdown PC9‐BrM3 cells with the ferroptosis inhibitor ferrostatin‐1 (Fer‐1) after the platinum treatments. Subsequently, we assessed the cell viability, irons concentration and lipid peroxidation by MDA (the end product of lipid peroxidation) kits and BODIPY™ 581/591 C11 (lipid peroxidation sensor).^21^, ^39^, ^40^ As expected, cell viability was remarkably restored in the experimental groups in which GPX4 or GSTM1 knockdown cells were cotreated with Fer‐1 and platinum drugs (Figure 4E‐F); in addition, the ferroptosis reaction was significantly inhibited in these groups (Figure 4G‐L), compared to the groups treated with platinum alone. In addition, we pretreated the PC9‐BrM3 cells with the GPX4 inhibitor (1S, 3R)‐RSL3 (widely used as a classical ferroptosis inducer ^41^) for 24 hours to pharmacologically inhibit the activity of GPX4, and then administered the same treatments as described above. We found that exogenous intervention of GPX4 also significantly improved the drug sensitivity of BM cells with obvious activation of ferroptosis; this phenomenon was reversed by additional Fer‐1 treatment (Figure S3G‐J). Collectively, these findings indicated that the suppression of GPX4 and GSTM1 recovered the platinum sensitivity of BM cells by inducing ferroptosis. Moreover, the results suggest that GSTM1 may be a novel negative regulator of ferroptosis in addition to GPX4.

### 3.5 GPX4 regulated the level of GSTM1 by protein stabilization

**FIGURE 5:**
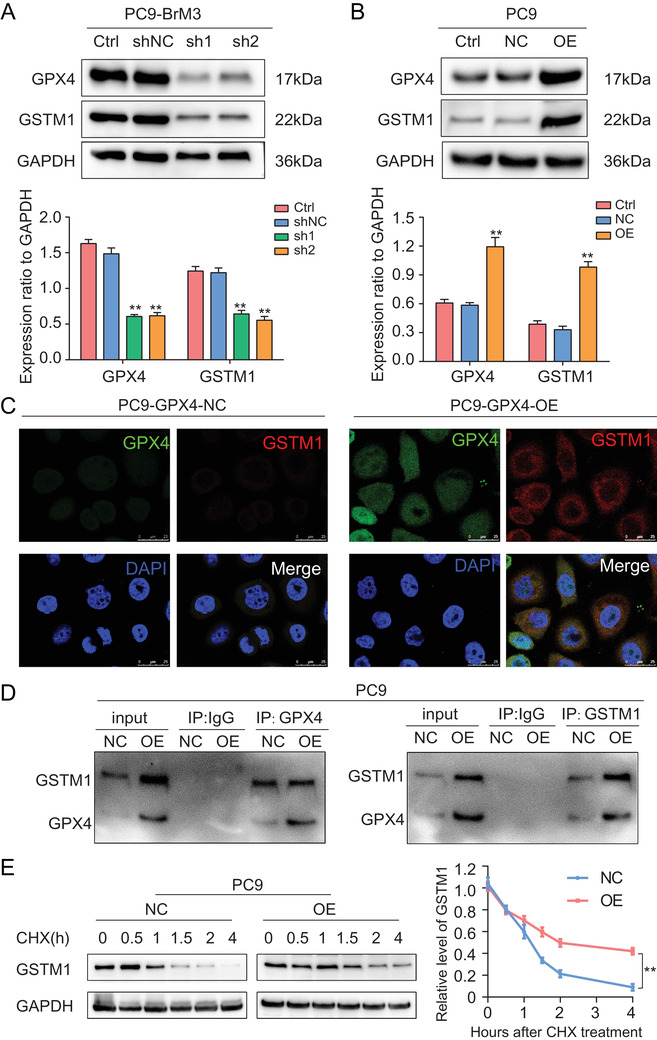
GPX4 regulated the level of GSTM1 by protein stabilization. (A) Representative Western blot image showing the GPX4 and GSTM1 expression in GPX4 knockdown PC9‐BrM3 cells. Ctrl, control PC9‐BrM3 cells; shNC, PC9‐BrM3 cells transfected with negative control shRNA; sh1, PC9‐BrM3 cells transfected with GPX4‐targeted shRNA vector 1; sh2, PC9‐BrM3 cells transfected with GPX4‐targeted shRNA vector 2. The bar graphs are summarized results from three independent experiments. **P < 0.01 versus shNC group. (B) Representative Western blot image showing the GPX4 and GSTM1 expression in PC9 cells with GPX4 overexpression. Ctrl, control PC9 cells; NC, PC9 transfected with negative control plasmid; OE, PC9 transfected with GPX4 plasmid. The bar graphs are summarized results from three independent experiments. **P < 0.01 versus NC group. (C) Immunofluorescent analysis of GPX4 (green) and GSTM1 (red) expression and distribution in PC9 cells with GPX4 overexpression. Cell nuclei are stained with DAPI (blue). Scale bar, 25 μm. NC, PC9 transfected with negative control plasmid; OE, PC9 transfected with GPX4 plasmid. (D) The potential interaction between GPX4 and GSTM1 was determined by immunoprecipitation using anti‐GPX4 and anti‐GSTM1 in PC9 cells with GPX4 overexpression. GPX4 was pulled down by anti‐GPX4, and then GPX4 and GSTM1 were detected by Western blot. On the contrary, GSTM1 was pulled down by anti‐GSTM1, and then GPX4 and GSTM1 were detected by Western blot. NC, PC9 transfected with negative control plasmid; OE, PC9 transfected with GPX4 plasmid. (E) Results of Western blot analysis showing protein expressions of GSTM1 in individual groups of cells following treatment with CHX (50 μM). The standardized quantitative curves show the protein degradation rate in the indicated groups. NC, PC9 transfected with negative control plasmid; OE, PC9 transfected with GPX4 plasmid (**P < 0.01). Representative images of each group from three independent experiments are presented

Since GPX4 and GSTM1 are concomitantly involved in glutathione metabolism and promoted drug resistance, we explored the potential interactions between the two. The protein‐protein interaction (PPI) network created by STRING predicted potential interaction between GPX4 and GSTM1 and their coexpression (Figure S4A). Indeed, we found significant reduction in the protein level of GSTM1 following the knock‐down of GPX4 in PC9‐BrM3 cells; conversely, the protein level of GSTM1 was upregulated following the overexpression of GPX4 in PC9 cells (Figure 5A‐B); however, there were no rules that GPX4 could be regulated with the change in GSTM1 expression. In addition, there was no significant difference in GSTM1 mRNA levels following the intervention of GPX4 (Figure S4B‐C).

To further investigate the mechanisms underlying the regulation of GSTM1 expression by GPX4, we colocalized the two proteins using confocal microscopy. We found coexpression of the two proteins in the cytoplasm. Moreover, the protein abundance of GSTM1 was significantly increased in the cytoplasm, but not in the nucleus, after induction of GXP4 overexpression (Figure 5C); this suggested that GSTM1 was regulated at the posttranscriptional level. In the coimmunoprecipitation assays, anti‐GPX4 antibody precipitated GSTM1, while anti‐GSTM1 also precipitated GPX4 in PC9 with stable overexpression of GPX4 cells (Figure 5D). This provided strong evidence of the direct interaction between GPX4 and GSTM1. Interaction between proteins, which plays an important role in the posttranslational modification (PTM) of proteins, has a wide spectrum of consequences for protein stability and the transactivation function.^42^, ^43^, ^44^ Here we explored whether GPX4 regulates the stabilization of GSTM1 protein. After treatment with cycloheximide (CHX), which is commonly used to determine the half‐life of proteins due to its ability to inhibit the synthesis of eukaryotic proteins, the relative levels of GSTM1 in GPX4 overexpressing PC9 cells were more stable compared to that in the control PC9 cells with low GPX4 expression (Figure 5E). These results suggested that GPX4 interacts and regulates the level of GSTM1 by enhancing the protein stability.

### 3.6 Wnt/NR2F2 signaling is responsible for transcriptional upregulation of GPX4 in BM

**FIGURE 6:**
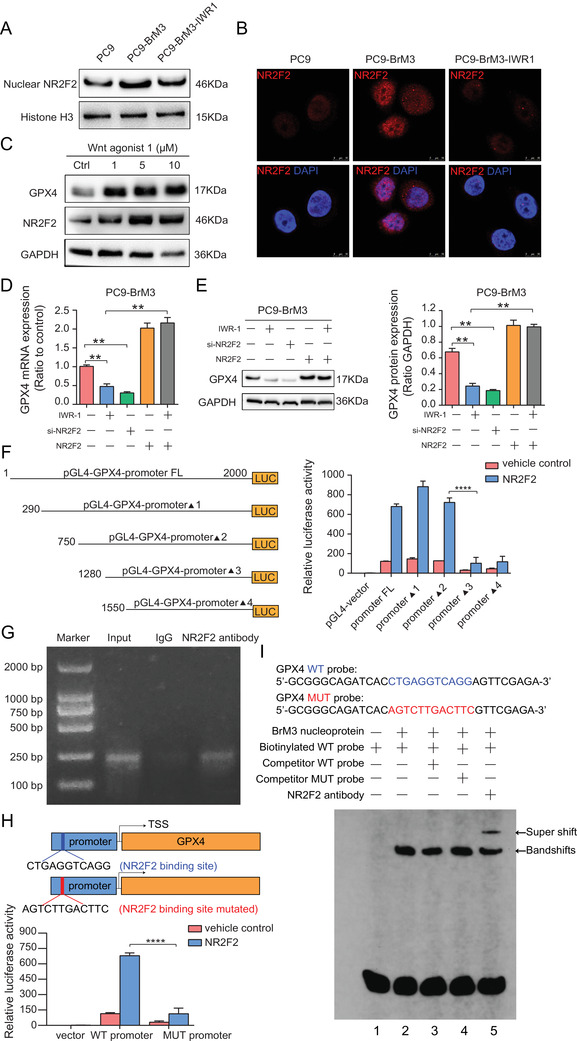
Wnt/NR2f2 signaling is responsible for transcriptional upregulation of GPX4 in BM. (A) Representative Western blot image showing the nuclear NR2F2 expression in PC9, PC9‐BrM3 cells, and PC9‐BrM3 cells treated with Wnt inhibitor IWR‐1‐endo for 48 hours. Histone H3 acted as the internal reference protein for nuclear protein. (B) Representative immunofluorescence staining images show the expression and location of NR2F2 in PC9, PC9‐BrM3 cells, and PC9‐BrM3 cells treated with Wnt inhibitor IWR‐1‐endo for 48 hours. Cell nuclei were stained with DAPI (blue) in PC9 cells with GPX4 overexpression (scale bar, 25 μm). (C) Results of Western blot showing the expressions of NR2F2 and GPX4 in PC9 cells treated with Wnt agonist 1 (1, 5, 10 μM) for 48 hours. Representative images of each group from three independent experiments are presented. Results of qPCR (D) and Western blot (E) showing the mRNA and protein expressions of GPX4 in PC9‐BrM3 cells after treatment with the Wnt inhibitor IWR‐1‐endo (10 μM) with or without transfection with NR2F2 plasmid, or transfected with NR2F2 siRNA or NR2F2 plasmid alone for 48 hours. Representative images of each group are presented and the bar graphs are summarized results from three independent experiments (*n* = 3, **P < 0.01). (F) Schematic illustration of a series of incremental deletion structures of the luciferase reporter gene (left) and the results of luciferase assays (right) where 293T cells were transiently transfected with the indicated pGL4 basic‐based reporter constructs for 24 hours and then the luciferase activity was measured (*n* = 3, ****P < 0.0001). (G) ChIP assay of NR2F2 binding to the GPX4 promoter. Lane 1, DNA marker; lane 2, input DNA; lane 3, DNA from PC9‐BrM3 cells immunoprecipitated with normal IgG; lane 4, DNA from PC9‐BrM3 cells immunoprecipitated with anti‐NR2F2 antibody. Representative images of each group from three independent experiments are presented. (H) Schematic illustration of the specific NR2F2 binding sites of GPX4 promotor (WT promoter) and the mutant sites (MUT promoter) for dual‐luciferase assays (upper). Results of luciferase assays (lower): 293T cells were transiently transfected with the indicated pGL4 basic‐based reporter constructs for 24 hours and then the luciferase activity was measured (*n* = 3, ****P < 0.0001). (I) Results of EMSA and supershift assay of NR2F2 binding to GPX4 promoter

After identification of the central role of GPX4 in regulating platinum‐based chemotherapeutic resistance in BM, we investigated the underlying mechanism of GPX4 regulation. The results of qPCR showed significantly higher mRNA level of GPX4 in PC9‐BrM3 as compared to that in PC9 cells (Figure S5A); this suggested that GPX4 was upregulated at the transcriptional level. Hyperactivation of canonical Wnt signaling has been shown to be a unique characteristic of highly brain metastatic subpopulations derived from human lung adenocarcinoma cell lines.^24^ We demonstrated that IWR‐1‐endo (synonyms: IWR‐1, a tankyrase inhibitor which inhibits canonical Wnt pathway) suppressed the expression level of GPX4 (Figure S5B); this indicated a correlation between GPX4 and canonical Wnt pathway. Transcription factors play a vital role in regulating the expression of protein‐coding genes on a genome scale by sequence‐specific binding to chromatin; in addition, these are usually mobilized by activation of specific signaling pathways.^45^, ^46^ To identify the potential transcription factor for GPX4, a series of candidates (NR2F2, KLF4, HNF4A, HNF4G, etc) were predicted by the PROMO database. Among these candidates, we prioritized the one that was significantly different in BM cells. A previous study performed RNA‐seq in PC9‐BrM3;^24^ thus, we mapped the protein profiling generated by our proteomic, cross‐aligning the gene expression profiling data of the RNA‐sEquation (GSE14107). We found that the NR2F2 (nuclear receptor subfamily 2, group F, member 2), which belongs to the nuclear receptor family of transcriptional regulators and possesses transcription factor activity,^47^, ^48^ was upregulated in PC9‐BrM3 compared with parental PC9, both in proteomics and in RNA‐seq. We first verified the increased nuclear expression of NR2F2 in PC9‐BrM3 compared with PC9 cells and its reduction after treatment with IWR‐1‐endo (Figures 6A‐B, S5C), while Wnt agonist 1 induced increased expressions of NR2F2 and GPX4 in PC9 cells (Figures 6C, S5D); these findings confirmed the activation of NR2F2 in BM cells and demonstrated the regulatory connection between the Wnt pathway and NR2F2.

To further verify the involvement of Wnt/NR2F2 signaling axis in controlling the transcription and expression of GPX4, PC9‐BrM3 cells were treated with IWR‐1‐endo, or NR2F2 siRNA interference, or NR2F2 plasmid, or cotreated with IWR‐1‐endo and NR2F2 plasmid while PC9 cells were treated with Wnt agonist 1, or NR2F2 plasmid, or NR2F2 siRNA interference, or cotreated with Wnt agonist 1 and NR2F2 siRNA interference; subsequently, the mRNA and protein expressions of GPX4 were measured. The results showed that both the protein and mRNA expressions of GPX4 were significantly regulated by Wnt/NR2F2 signaling (Figures 6D‐E, S5E‐F). The platinum sensitivity of cells showed a similar trend to that of GPX4 expression under the above‐mentioned interventions of NR2F2 and Wnt pathway, either in PC9‐BrM3 or PC9 cells (Figure S5G‐H); this suggested that GPX4 was regulated by the Wnt/NR2F2 signaling axis and that Wnt/NR2F2/GPX4 contributed to the acquired drug resistance. We further determined the transcriptional region of the GPX4 gene promoter that is responsive to NR2F2. We introduced a series of pGL4 basic‐based luciferase reporter constructs with incremental deletions within 2000 bp upstream of the GXP4 transcription start site (TSS) into 293T cells. Luciferase reporter assays indicated that the region between 750 and 1280 bp upstream of the GXP4 TSS is required for the transcriptional upregulation of GXP4 by NR2F2, providing convincing clues for presuming the binding sites (Figure 6F). Further chromatin immunoprecipitation verified the direct binding of NR2F2 to the promoter of GPX4 (Figure 6G). To confirm that NR2F2 binds to the presumed sites of GPX4 promoter in PC9‐BrM3 cells, we induced a mutation in the predicted binding site and then performed dual‐luciferase assay (Figure 6H). Moreover, we synthesized oligonucleotides covering the presumed sites and mutated sites as a WT probe and a MUT probe respectively, and applied these in EMSA experiments (Figure 6I). These results suggested that NR2F2 specifically binds to GPX4 promoter (Figure 6H‐I). Collectively, these results demonstrated that Wnt/NR2F2 is directly responsible for the transcriptional upregulation of GPX4 in BM.

### 3.7 GPX4 targeting inhibitor therapeutically enhances the anticancer activity of platinum in vivo

**FIGURE 7:**
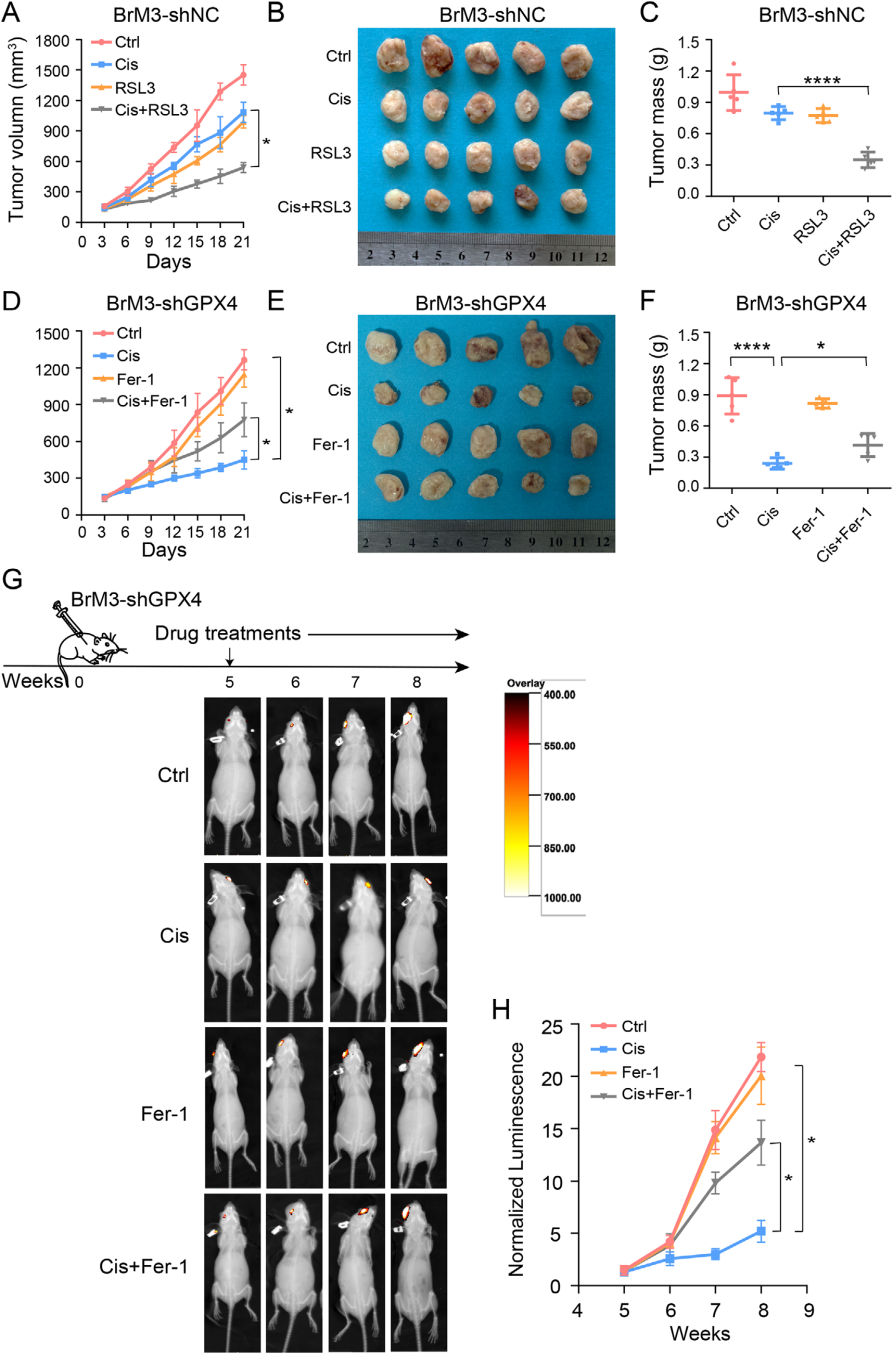
GPX4 targeting inhibitor therapeutically enhances the anticancer activity of platinum in vivo. Nude mice were injected subcutaneously with PC9‐BrM3 cells with negative control shRNA transfection (BrM3‐shNC) (5×10^6^ cells/mouse) and treated with DMSO as control (Ctrl, *n* = 5, once every 5 days, i.p.) or 5 mg/kg cisplatin (Cis, *n* = 5, once every 5 days, i.p.) with or without 100 mg/kg RSL3 (*n* = 5 per group, twice a week, s.c. at tumor site) while another five mice were administered RSL3 alone. Tumor size was measured every 3 days using caliper to the plot growth curve (A); tumor mass was weighed at the end of the experiment (B and C) (*P < 0.05, ****P < 0.0001). Nude mice were injected subcutaneously with PC9‐BrM3 cells with GPX4 shRNA transfection (BrM3‐shGPX4) (5×10^6^ cells/mouse) and treated with DMSO as control (Ctrl, *n* = 5, once every 5 days, i.p.) or 5 mg/kg cisplatin (Cis, *n* = 5, once every 5 days, i.p.) with or without 0.2 mg/kg ferrostatin‐1 (Fer‐1, *n* = 5 per group, daily, i.p.) while another five mice were administered ferrostatin‐1 alone. Tumor size was measured every 3 days using caliper to plot growth curve (D); tumor mass was weighed at the end of experiment (E and F) (*P < 0.05, ****P < 0.0001). (G) Heat map image representations of bioluminescence intensity for representative mice from the indicated groups of the therapy response experiment. Nude mice were implanted with PC9‐BrM3 cells with GPX4 shRNA transfection (BrM3‐shGPX4) (10^6^ cells/mouse) by intracardiac injection. Treatments were initiated after 4 weeks. Mice were treated with DMSO as control (Ctrl, *n* = 3, once every 5 days, i.p.) or 5 mg/kg cisplatin (Cis, *n* = 3, once every 5 days, i.p.) with or without 0.2 mg/kg ferrostatin‐1 (Fer‐1, *n* = 3 per group, daily, i.p.) while another three mice were administered ferrostatin‐1 alone. Bioluminescence intensity in the same bioluminescence heat map range was measured every week. (H) Plot of mean bioluminescence readings for control and treatment group mice; the standard error is indicated for each imaging point (*n* = 3, *P < 0.05)

(1S, 3R)‐RSL3 is a known GPX4 inhibitor which directly binds to GPX4 and inactivates its peroxidase activity.^22^ However, the poor systemic bioavailability of RSL3 limits its use and it can only be used for subcutaneous xenograft tumors in nude mice by direct intratumoral injection.^22^, ^49^ To determine whether RSL3 improves the anticancer effect of platinum in vivo, we administered the tumor, which was formed by subcutaneous injection of PC9‐BrM3 with the control shRNA (BrM3‐shNC) in nude mice, in combination with cisplatin and RSL3. The results showed that RSL3 remarkably enhanced the antitumor effect of cisplatin while either RSL3 or cisplatin alone had negligible effects on the in vivo growth of BrM3‐shNC (Figure 7A‐C). To further test GPX4 dependence of tumor platinum sensitivity in vivo, we also genetically suppressed the expression of GPX4 by shGPX4 transfection and implanted GPX4 knockdown PC9‐BrM3 cells (BrM3‐shGPX4) into nude mice by subcutaneous and intracardiac injection, respectively. Consistent with the previous findings, BrM3‐shGPX4 was remarkably sensitive to cisplatin in vivo and the sensitivity was restored by cotreatment with ferroptosis inhibitor (Fer‐1), both in the subcutaneous xenograft models and in the spontaneous BM models (Figure 7D‐H); further, the obtained subcutaneous masses were stained for GPX4 to confirm the knock‐down efficiency (Figure S6A,C), while intratumor total iron concentrations were assessed to determine ferroptosis in various groups (Figure S6B,D). This further confirmed the involvement of ferroptosis in the underlying mechanism.

Collectively, these data indicated that inhibition of GPX4 rendered the brain metastatic cells more responsive to cisplatin via induction of ferroptosis in vivo.

## 4 DISCUSSION

Platinum‐based chemotherapy has been shown to confer survival benefit in patients with lung cancer; however, it shows limited therapeutic efficacy against BM. Two main factors are believed to account for the ineffectiveness of chemotherapy against BM: the existence of blood‐brain barrier (BBB) and the endogenous intratumor changes caused by metastatic events. BBB prevents the entry of foreign substances in the brain and offers a refuge site for tumors to evade drug therapies. However, recent studies have demonstrated disruption of the barrier function of the BBB in the setting of BM, which allows tumor cells to invade and colonize the brain parenchyma.^25^, ^50^ This indicates that the barrier blocks the entrance of drugs is compromised in the setting of BM. This supports the view that autologous factors of metastatic cells may have a major part in the acquisition of chemotherapeutic resistance.

In this study, we established a series of BM subpopulations (PC9‐BrMs) from the parental cells PC9 and confirmed the obvious resistance of brain metastatic cells to platinum. This highlights the need to explore the endogenous changes in BM cells. Therefore, metabolomics and proteomics were jointly employed to reveal differential metabolic and protein expression profiles of BM subpopulations compared to parental cells. The metabolomics study revealed a greatly altered metabolic state of BM; the most prominent change was the active oxidative stress characterized by high ROS accumulation and the subsequent antioxidant metabolic response. Studies have indicated that the metastasizing cancer cells indeed experience heightened oxidative stress in distant organs. Incompatible brain microenvironment (higher O_2_ tension, different mesenchymal cell, neuroimmune response, etc) contributed to the high thresholds of ROS, which may trigger cell senescence, apoptosis, or ferroptosis. To survive and form metastatic lesions in a harsh milieu, metastatic cells increase their antioxidant status by reprogramming the metabolism and protein expression.^51^, ^52^, ^53^ Consistently, omics performed by us comprehensively revealed the response of BM subpopulations under these condition; the response included metabolic reprogramming, like the hyperactive pentose phosphate pathway (PPP) and GSH metabolism, which play an important role in the biosynthesis of cellular reductant, and regulation of protein expressions, like the upregulated GPX4 and GSTM1 which were demonstrated to render the BM cells tolerant to ferroptosis triggered by lipid ROS by consuming GSH. GSH, which plays a vital role in cellular oxidative stress with its antioxidant properties, helps cancer cells to minimize oxidative stress, during which process GSH is consumed and converted to its oxidized form (GSSG).^54^ These findings provide a plausible explanation of the increased consumption of GSH in brain metastatic cells. We hypothesized that the increased GSH consumption may facilitate the BM process as well as the acquisition of drug resistance. Extracellular cystine is reduced to cysteine intracellularly, and the uptake of cystine is rate‐limiting for the synthesis of GSH;^33^, ^34^, ^35^ therefore, extracellular cystine is a sensitive indicator of GSH status. The metabolomics study indicated a unique metabolic status wherein GSH was highly consumed; therefore, we further determined and compared the levels of cystine in the serum of lung cancer patients with and without BM by GC‐MS; the results confirmed that the unique metabolic status was common for BM and merits further investigation.

The results of proteomics revealed the underlying reasons for the metabolic changes in BM. The depletion of GSH was found to have been caused by the upregulation of GPX4 and GSTM1. We confirmed that the upregulation of GPX4 and GSTM1 increased the resistance of BM cells to platinum‐based chemotherapy by inhibiting ferroptosis. We identified that GSTM1 may be a novel regulator of ferroptosis in addition to GPX4 (a well‐known regulator for ferroptosis). GSTM1 is a member of glutathione S‐transferases which catalyzes the conjugation of GSH to a wide range of compounds. It is hypothesized that GSTM1 catalyzes the conjugation of GSH to the oxidized products of lipid peroxidation to suppress ferroptosis;^22^, ^56^, ^57^, ^58^ however, further studies are required to confirm this hypothesis. Besides, we also found that GSTM1 in BM cells was regulated posttranslationally by GPX4; however, the specific protein interactions and modifications have not yet been clarified. Therefore, further in‐depth studies are warranted to clarify this aspect.

Our findings indicated a central role of GPX4 in mediating platinum resistance in lung cancer BM cells. Further we determined the correlation between the levels of cystine and GPX4 in lung cancer patients with BM; this suggests a close relationship between the metabolism of GPX4 and GSH in BM. Our findings provide new insights into the application of concomitant detection of cystine and GPX4 in clinical settings to assess the chemotherapeutic response of lung cancer patients with BM. We also traced the mechanism of upregulation of GPX4 in BM and identified the Wnt/NR2F2 pathway as a determinant of transcriptional upregulation of GPX4 in BM. The Wnt signaling pathway has a major part in stem cell differentiation, embryonic development, tumorigenesis, and tumor progression.^59^ Studies have highlighted the vital role of activation of canonical WNT pathway in determining the occurrence of brain metastases in the setting of lung adenocarcinoma progression.^24^ Hyperactive Wnt signaling induces a series of signal transduction leading to accumulation of β‐catenin in the nucleus and its binding to transcription factors to regulate gene expression.^60^ NR2F2 is one of the transcription factors that can be activated by the Wnt signaling pathway.^61^ We confirmed increased expression of NR2F2 in the nucleus of BM cells, which was inhibited after treatment with Wnt inhibitor, and caused subsequent promotion of GPX4 transcription, suggesting that GPX4 is a target gene of NR2F2. We further explored the specific sites where NR2F2 acts as a transcription factor by binding to the promoter region of the target gene *GPX4*; the results showed that Wnt/NR2F2 was responsible for the overexpression of GPX4 in BM cells and that the Wnt/NR2F2/GPX4 axis promoted the acquisition of chemoresistance in BM. Upregulation of Wnt signaling has been shown to induce resistance to a variety of traditional and targeted cancer treatments via multiple mechanisms, including by maintaining the number of cancer stem cells, enhancing DNA damage repair, promoting transcriptional plasticity, and promoting immune escape.^62^, ^63^ Moreover, GPX4 has been identified as a critical survival protein for cancer cells in a high mesenchymal therapy‐resistant cell state in the antilipid peroxidase pathway ^14^, ^23^ while there is a paucity of studies investigating the effects of NR2F2 on drug resistance. However, this is the first study to investigate whether the transcriptional regulation of GPX4 is via the Wnt/NR2F2 axis or its promoting role in the acquisition of chemoresistance by lung cancer BM. This highlights the novelty of the present study, which suggests that blockade of Wnt/NR2F2/GPX4 axis may serve as a novel therapeutic strategy for improving the effect of chemotherapy in lung cancer BM.

Moreover, GPX4 is an important selenoprotein for cellular survival and selenium has been regarded as an essential micronutrient that has a broad influence on human health and disease including cancers.^64^ Cohort studies have indicated a protective effect of selenium against lung cancer in populations where average selenium levels are low, although selenium is not currently considered as a general strategy for lung cancer prevention.^65^ Recent studies suggest that selenium utilization by GPX4 is necessary for inhibiting ferroptosis, highlighting the important effects of selenium on GPX4 activity.^66^, ^67^ Here we demonstrate that GPX4 drives platinum chemoresistance in lung cancer‐derived BM by suppressing ferroptosis, indicating the potential role of selenium in promoting chemotherapy sensitivity in lung cancer patients with BM. However, further well‐organized and intensive prospective clinical studies are required to analyze the correlation between total dietary selenium intake, GPX4 activity, chemotherapy response, and survival outcomes, as well as other information such as medication history like statins, whose usage may cause impaired selenoprotein expression in patients with lung cancer with BM.^67^

## 5 CONCLUSIONS

**FIGURE 8:**
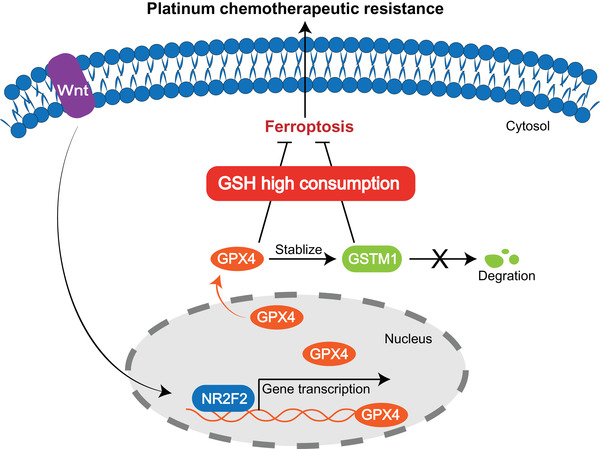
Illustration of the potential mechanisms by which GPX4 induces platinum chemotherapeutic resistance in lung cancer brain metastasis. In brain metastatic lung cancer cells, hyperactive Wnt pathway motives the transcription factor NR2F2 binding to the promoter of target gene GPX4, promoting the transcription of GPX4. GPX4 stabilizes and posttranslationally regulates the level of GSTM1. Upregulation of both GPX4 and GSTM1 inhibits ferroptosis characterized with lipid peroxidation at the cost of high consumption of glutathione (GSH), leading to the chemotherapeutic resistance to platinum drugs

In summary, GPX4, transcriptionally activated by Wnt/ NR2F2 pathway, is at the heart of glutathione metabolism and ferroptosis, mediating platinum‐based resistance in BM. On the one hand, GPX4 directly suppresses lipid peroxidation by catalyzing the oxidation of GSH. On the other hand, GPX4 posttranslationally regulates the level of GSTM1, which also acts as a negative regulator of ferroptosis and is involved in glutathione metabolism by catalyzing the covalent binding of GSH to oxidized products of lipid peroxidation (Figure 8). Application of GXP4 targeting inhibitor enhanced the response of BM cells to platinum chemotherapy in vivo, presenting a novel strategy to overcome chemotherapeutic resistance in lung cancer BM.

## ETHICS APPROVAL AND CONSENT TO PARTICIPATE

Clinical studies were approved by the Ethics Review Committee of the Second Hospital of Dalian Medical University and animal studies were approved by the Dalian Medical University Licensing Committee.

## AUTHOR CONTRIBUTIONS

WL and YZ designed, performed, and analyzed experiments, performed bioinformatic analyses, and contributed to writing the manuscript. WL and WD performed experiments. JS analyzed data. SX, XD, and SW processed clinical samples and provided clinical information. EL and CR provided protocols and technical input. QW and WW conceived and supervised the project, designed, and analyzed experiments, and revise the manuscript. All authors read and approved the submitted manuscript.

## CONFLICT OF INTEREST

The authors have declared that no conflict of interest exists.

## Supporting information



Supporting Information

## Data Availability

The mass spectrometry proteomics and metabolomics data have been deposited to the ProteomeXchange Consortium (http://proteomecentral.proteomexchange.org/cgi/GetDataset) via the PRIDE partner repository with the dataset identifier PXD019571 (proteomics data, username: reviewer28700@ebi.ac.uk, password: PVbsJtqQ) and PXD019638 (metabolomics data, username: reviewer84586@ebi.ac.uk, password: WnwLRMHB)
