# Supplementary material for: Glutathione peroxidase 4‐dependent glutathione high‐consumption drives acquired platinum chemoresistance in lung cancer‐derived brain metastasis
Source: Clin Transl Med. 2021 Sep 26;11(9):e517. doi: 10.1002/ctm2.517 (PMC8473645; doi:10.1002/ctm2.517)
Supplement: Supplementary file 1 — Supporting Information [file CTM2-11-e517-s001.docx]

**Materials and methods**

1. Quantitative tandem mass tag (TMT)-based proteomics
   1. Protein Extraction

Cells was harvested and sonicated three times on ice using a high intensity ultrasonic processor (Scientz) in lysis buffer (8 M urea, 1% Protease Inhibitor Cocktail). The remaining debris was removed by centrifugation at 12,000 g at 4 °C for 10 min. Finally, the supernatant was collected and the protein concentration was determined with BCA kit (Thermo Fisher Scientific Inc., USA) according to the manufacturer’s instructions.

- 1. Trypsin Digestion

For digestion, the protein solution was reduced with 5 mM dithiothreitol for 30 min at 56 °C and alkylated with 11 mM iodoacetamide for 15 min at room temperature in darkness. The protein sample was then diluted by adding 100 mM TEAB to urea concentration less than 2M. Finally, trypsin was added at 1:50 trypsin-to-protein mass ratio for the first digestion overnight and 1:100 trypsin-to-protein mass ratio for a second 4 h-digestion.

1.3 TMT Labeling

After trypsin digestion, peptide was desalted by Strata X C18 SPE column (Phenomenex) and vacuum-dried. Peptide was reconstituted in 0.5 M TEAB and processed according to the manufacturer’s protocol for TMT kit. Briefly, one unit of TMT reagent was thawed and reconstituted in acetonitrile. The peptide mixtures were then incubated for 2 h at room temperature and pooled, desalted and dried by vacuum centrifugation.

- 1. HPLC Fractionation

The tryptic peptides were fractionated into fractions by high pH reverse-phase HPLC using Agilent 300Extend C18 column (5 μm particles, 4.6 mm ID, 250 mm length). Briefly, peptides were first separated with a gradient of 8% to 32% acetonitrile (pH 9.0) over 60 min into 60 fractions. Then, the peptides were combined into 18 fractions and dried by vacuum centrifuging.

1.5 LC-MS/MS Analysis

The tryptic peptides were dissolved in 0.1% formic acid (solvent A), directly loaded onto a home-made reversed-phase analytical column (15-cm length, 75 μm i.d.). The gradient was comprised of an increase from 6% to 23% solvent B (0.1% formic acid in 98% acetonitrile) over 26 min, 23% to 35% in 8 min and climbing to 80% in 3 min then holding at 80% for the last 3 min, all at a constant flow rate of 400 nL/min on an EASY-nLC 1000 UPLC system.

The peptides were subjected to NSI source followed by tandem mass spectrometry (MS/MS) in Q ExactiveTM Plus (Thermo) coupled online to the UPLC. The electrospray voltage applied was 2.0 kV. The m/z scan range was 350 to 1800 for full scan, and intact peptides were detected in the Orbitrap at a resolution of 70,000. Peptides were then selected for MS/MS using NCE setting as 28 and the fragments were detected in the Orbitrap at a resolution of 17,500. A data-dependent procedure that alternated between one MS scan followed by 20 MS/MS scans with 15.0s dynamic exclusion. Automatic gain control (AGC) was set at 5E4. Fixed first mass was set as 100 m/z.

- 1. Database Search

The secondary mass spectrometry data was retrieved using Maxquant (v1.5.2.8). Retrieval parameter settings: the database is SwissProt Human (20317 sequences), an anti-library was added to calculate the false positive rate (FDR) caused by random matching, and a common pollution library was added to the database to eliminate contaminated proteins in identification The effect of enzyme digestion was set to Trypsin / P; the number of missed cleavage sites was set to 2; the minimum length of peptides was set to 7 amino acid residues; the maximum number of modifications of peptides was set to 5; the ion mass error tolerance was set to 20 ppm and 5 ppm, respectively, and the mass error tolerance of the secondary fragment ion was 0.02 Da. The cysteine alkylation was set as a fixed modification, and the variable modification was the oxidation of methionine, the acetylation of the N-terminus of the protein, and the deamidation of asparagine and glutamine. The quantitative method was set to TMT-6plex, and the FDR for protein identification and PSM identification was set to 1%.

- 1. Bioinformatics Methods

The UniProt-GOA database (<http://www.ebi.ac.uk/GOA/>) gave Gene Ontology (GO) annotation. The KyotoEncyclopedia of Genes and Genomes (KEGG) database (<https://www.genome.jp/kegg/tool/map_pathway2.html>) identified enriched pathways. All identified differentially expressed proteins were entered into the KEGG database to search for related pathways. The output of the pathways was automatically classified into grade categories, and these grades are considered valid when the corrected p-value <0.05. DAVID (<https://david.ncifcrf.gov/>) served as a functional annotation tool to provide the enrichment results. The protein−protein interaction (PPI) network was created by STRING (<https://string-db.org/>) and PIPs (http://www.compbio.dundee.ac.uk/www-pips/). Then, import the text file with attributes into the open source software Cytoscape 3.5.2 (https://cytoscape.org/) for network visualization.

1. **Western Blot**

RIPA cell lysis buffer (MA0151, Meilunbio, China) containing a protease inhibitor cocktail and a phosphatase inhibitor cocktail (Sigma, USA) was used to dissolve proteins extracted from cells. The BCA assay kit (Thermo Fisher Scientific Inc., USA) was used to determine the protein concentration. Protein lysates were then separated by sodium dodecyl sulfate-polyacrylamide gel electrophoresis (SDS-PAGE) and transferred onto nitrocellulose membranes (Millipore, Billerica, USA). The membranes were blocked in 5% skimmed milk solution in 0.05% Tris-buffered saline/Tween-20 (TBST) and then incubated with primary antibodies against GPX4 (1:1,000 dilution; Abcam, UK), GSTM1 (1:1,000; Abcam, UK), GCLC (1:1,000; Proteintech, USA), xCT (1:1,000; Abcam, UK), glutathione synthetase (1:1,000; Abcam, UK) and GAPDH (1:5,000; Proteintech, USA). After washing with 0.05% TBST, the corresponding secondary antibodies conjugated with horseradish peroxidase (1:5,000; Proteintech, USA) were further used. The ECL western blotting substrate (NCM Biotech, China) was used to analyze the chemiluminescence of the blots. Protein expression was quantified by Image J software (National Institutes of Health, USA). The tests were performed with triplicated samples to diminish variability.

1. **Enzyme-linked immunosorbent assay (ELISA)**

GXP4 levels in the clinical serum were measured by sandwich ELISA using kits from Omnimabs (Omnimabs, California, USA). Briefly, the kit assay used purified antibody to coat microtiter plate wells to form solid-phase antibody. Samples, including standards, were pipetted onto coated microtiter wells. After incubation, biotinylated antibody and combined Streptavidin-HRP were added to form antibody-antigen-enzyme-antibody complex. After thoroughly washing, the tetramethylbenzidine (TMB) substrate solution was added. The color change was visualized after stopping the reaction with (NH_4_)_2_SO_4_. Change in color was measured at 450 nm with a microtiter plate reader, and the OD value was observed to be directly proportional to protein concentrations. Protein concentration was calculated by referencing to the standard curve.

1. **q-PCR (quantitative-polymerase chain reaction)**

Total RNA was extracted using the Trizol® reagent (Transgen biotech, China) and quantitated at OD_260_nm. Total RNA (1.0 μg) was treated with RNase-free DNase I and reverse-transcribed into cDNA using random primers and Superscript II^®^ retrotranscriptase (Invitrogen, USA). The resulting cDNAs were mixed with the SYBR PCR master mix (Applied Biosystems, USA) and run on the StepOnePlus Applied Biosystems Realtime PCR machine. One cycle of a denaturing step (3 min at 95 °C) was applied, which was followed by 40 cycles of amplification (12 s at 95 °C, 30s at 62 °C and 30s at 72°C), with fluorescence measured during the extension. Primers used in this study are as follows：

*GAPDH*: 5′-CATGAGAAGTATGACAACAGCCT-3′(forward);
5′-AGTCCTTCCACGATACCAAAGT-3′(reverse);

*GPX4*: 5′-TTTCCGCCAAGGACATC-3′(forward);
 5′-TTCCCGAACTGGTTACACG-3′(reverse).

*GSTM1*: 5′-GGGACGCTCCTGATTATGAC-3′(forward);
 5′-AGTAGGGCAGATTGGGAAAGT-3′(reverse).

The relative quantification value of mRNA expression was calculated using the comparative CT (^ΔΔ^CT) method and Step-OnePlus software v2.0.1 (Applied Biosystems, USA) with *GAPDH* as an internal control. Three independent experiments were performed to derive average relative quantification and standard deviation.

1. **Confocal immunofluorescence**

PC9 cells were cultured on the Glass Bottom Cell Culture Dish for 24 h and then were fixed with 4% paraformaldehyde, permeabilized with 0.25% of Triton X-100 (Solarbio), and treated with 5%

BSA. After being washed, the cells were probed with 1:100 diluted antibodies against GPX4 (1:100, Abcam) and GSTM1 (1:100, Santa Cruz Biotechnology) overnight at 4 °C. Subsequently, the cells were incubated with fluorophore-conjugated secondary antibodies (1:100, Proteintech) for 1 h and stained with DAPI (Sigma) for nuclei, followed by photoimaging under a confocal laser scanning microscope (Leica DM14000B).

1. **Small interfering RNA (siRNA), plasmid and short hairpin RNA (shRNA) transfection.**

The siRNA and plasmid for NR2F2, GPX4 and GSTM1 were designed and synthesized by GenePharma (Suzhou, China). The target sequences of NR2F2, GPX4 and GSTM1 plasmids were referred to its genetic sequence in the PubMed (https://www.ncbi.nlm.nih.gov/gene). The sequences of NR2F2 siRNA oligos were 5’-GGCCGUAUAUGGCAAUUCATT-3’ The siRNA oligos and plasmids were transfected by Lipofectamine 2000 (Invitrogen, Carlsbad, CA) following the manufacturer’s instructions.

The GPX4-targeting shRNA, GSTM1-targeting and its negative control shRNA (shNC) constructed in the LV16 lentiviral vector, and lentiviral particles expressing luciferase infused shRNA were packaged in 293T cells (GenePharma, Suzhou, China). The target sequences of GPX4 shRNA1 and shRNA2 were 5′-GCCUUUGCCGCCUACUGAATT-3′, and 5′-GGAGUAACGAAGAGAUCAATT-3′, respectively. The target sequences of GSTM1 shRNA1 and shRNA2 were 5′-GCCAAAGUACUUGGAGGAATT-3′, and 5′-UCUCGUCUAUGAUGUCCUUTT-3′, respectively. Infection of the shRNA-expressing lentivirus and establishment of stably infected single clones were carried out as directed by the manufacturer. The knockdown efficiency was evaluated by western blot analysis. Complete medium containing puromycin (1μg/ml) was used to screen the transfected positive cells and continuous culture to ensure stable gene expression in the cells.

1. **Immunohistochemistry (IHC) staining**

Briefly, tissue sections (3 µm) were deparaffinized, rehydrated, incubated with 3% H_2_O_2_ in methanol and subjected to antigen retrieval by EDTA buffer. The sections were blocked with 5% bovine serum albumin (BSA), probed with anti-GPX4 (1:100, Proteintech) at 4 °C overnight. The sections were reacted with biotinylated secondary antibodies, and detected using the Streptavidin-Peroxidase IHC assay kit and DAB (Solarbio, Beijing, China).

**Figures**
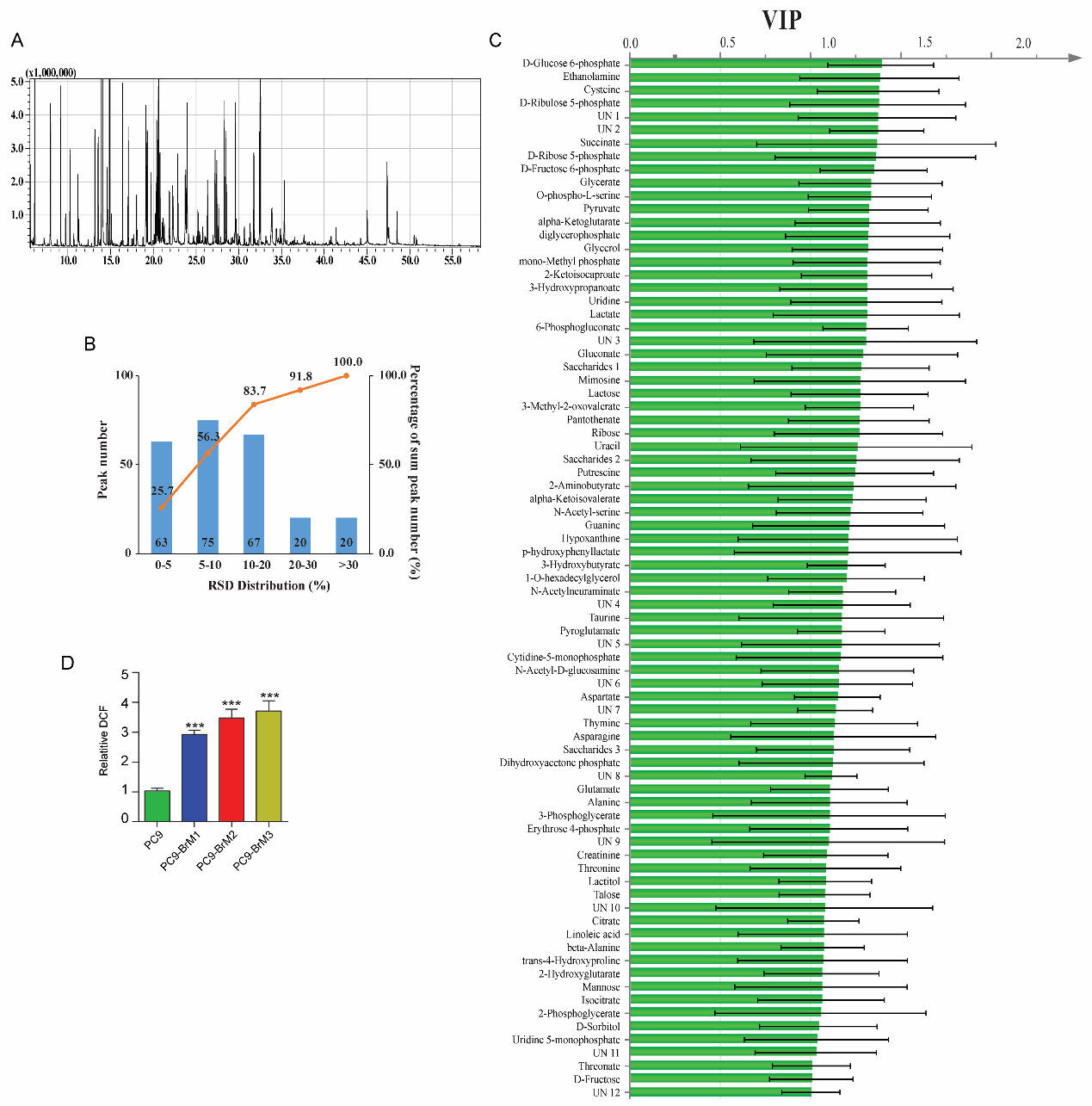


**Figure S1.** **Metabolic profiling in lung cancer BM.** (A) A typical total ion chromatogram of lung cancer BM cell metabolic profiling. (B) The RSD distribution of metabolic features in QC samples. (C) The column plot of VIP value with jack-knifed confidence intervals in PLS-DA model. (D) Reactive oxygen species levels (DCF stain) in cells. (n=3, ***p<0.001 versus PC9 group).


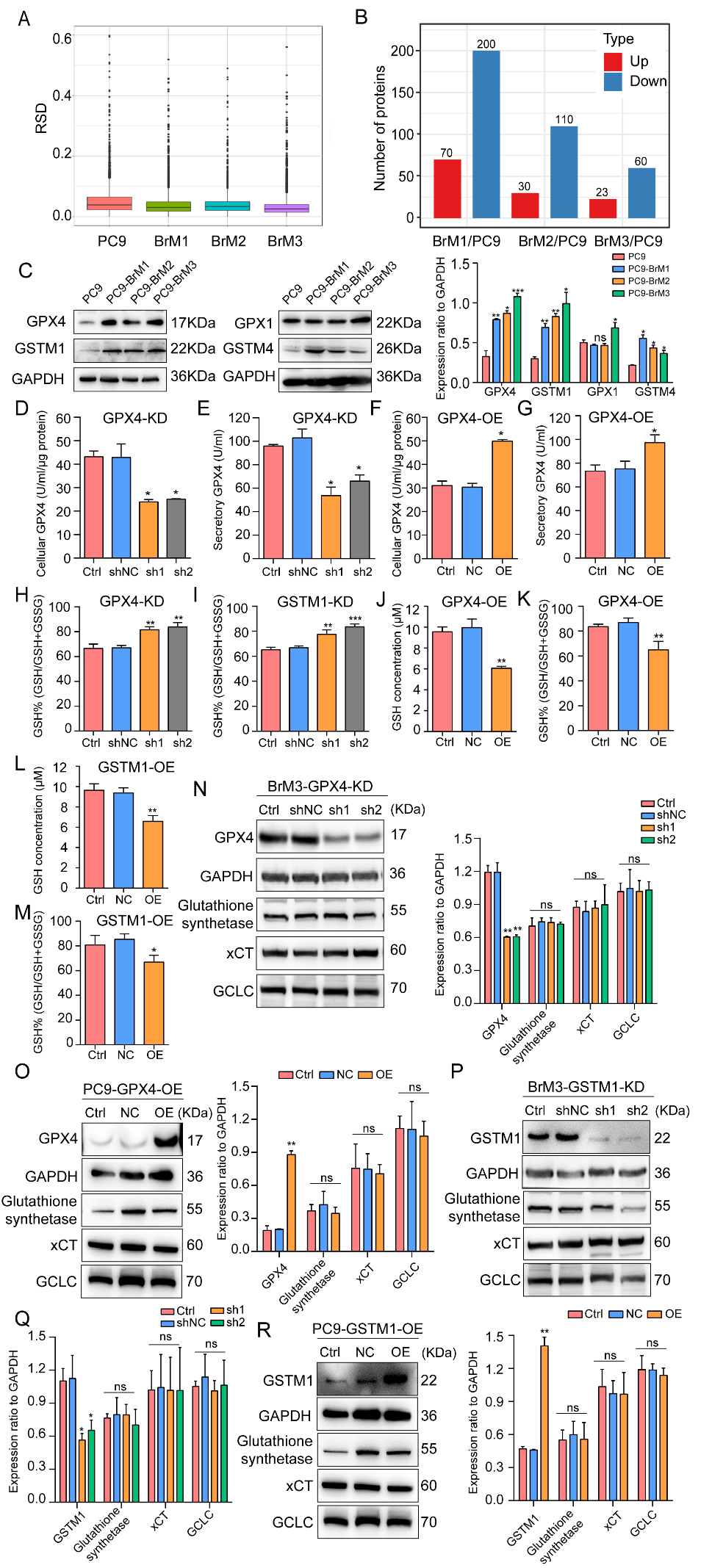


**Figure S2. Proteomics identified the role of GPX4 and GSTM1 in glutathione metabolism.** (A) The RSD distributions of QC samples in proteomics data. (B) Numbers of proteins identified in each subgroup with the ratio fold (BrM/PC9) greater than 1.5. (C) Results of Western blot analysis showing expressions of GPX4, GSTM1, GPX1 and GSTM4 in brain metastatic subpopulations. Representative images of each group are presented and the bar graphs are summarized results from 3 independent experiments. ns, no statistic difference; *p<0.01, **p<0.01, ***p<0.001, versus PC9 group. (D-G) ELISA results showing cellular or secretory GPX4 activities in PC9-BrM3 cells with GPX4 knocked down (KD) and in PC9 cells with GPX4 overexpressed (OE). Ctrl, control PC9-BrM3 or PC9 cells; shNC, PC9-BrM3 cells transfected with negative control shRNA; sh1, PC9-BrM3 cells transfected with GPX4-targeted shRNA-1; sh2, PC9-BrM3 cells transfected with GPX4-targeted shRNA-2; NC, PC9 cells transfected with negative control plasmid; OE, PC9 cells transfected with GPX4 plasmid. (n=3, *p<0.05, versus shNC or NC group) (H-I) The concentration of glutathione (GSH) with GPX4 or GSTM1 knocked down in PC9-BrM3 cells were assayed. The concentrations of glutathione (GSH) were assayed and evaluated by the ratio of GSH to the reduced plus oxidized glutathione (GSH+GSSG) levels. (n=3, **p<0.01, ***p<0.001, versus shNC group) Ctrl, control PC9-BrM3 cells; shNC, PC9-BrM3 cells transfected with negative control shRNA; shGPX4, PC9-BrM3 cells transfected with GPX4-targeted shRNA vector; shGSTM1, PC9-BrM3 cells transfected with GSTM1-targeted shRNA vector; sh1, PC9-BrM3 cells transfected with GPX4 or GSTM1-targeted shRNA-1; sh2, PC9-BrM3 cells transfected with GPX4 or GSTM1-targeted shRNA-2. (J-M) The level and concentration of GSH with GPX4 or GSTM1 overexpressed in PC9 cells were assayed. The concentrations of GSH were assayed and evaluated by the ratio of GSH to the reduced plus oxidized glutathione (GSH+GSSG) levels. (n=3, *p<0.05, **p<0.01, versus NC group) Ctrl, control PC9 cells; NC, PC9 cells transfected with negative control plasmid; OE, PC9 cells transfected with GPX4 or GSTM1 plasmid. (J-K) Results of Western blot analysis showing the expressions of GSH regulatory proteins or enzymes in indicated groups. Representative images of each group are presented and the bar graphs are summarized results from 3 independent experiments. GCLC, glutamate-cysteine ligase catalytic subunit; xCT, cystine/glutamate antiporter solute carrier family 7 member 11 (also known as SLC7A11). (ns, no statistic difference; *p<0.05, **p<0.01, versus shNC or NC group)


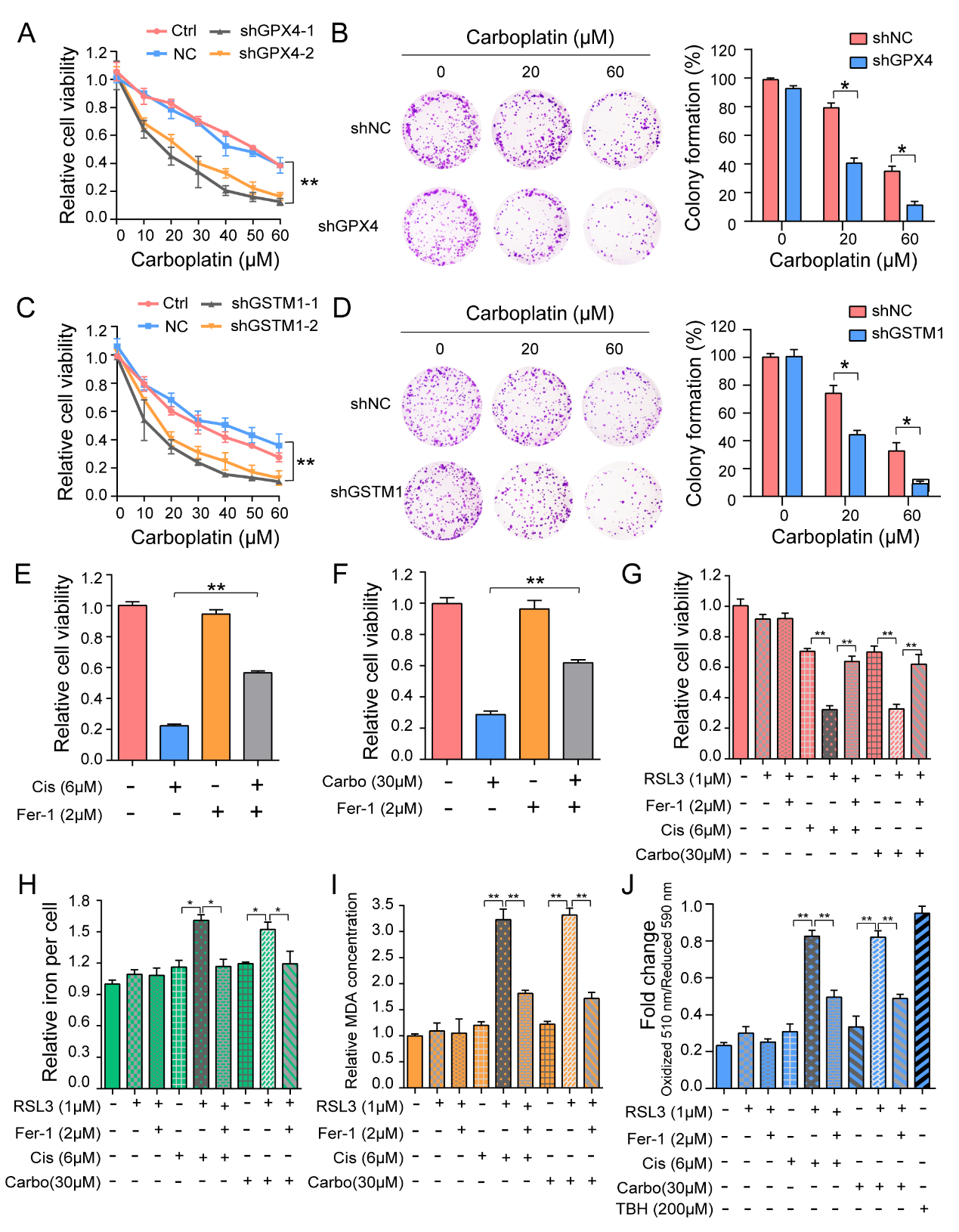


**Figure S3. Suppression of GPX4 and GSTM1 enhanced the platinum sensitivity of BM cells by inducing ferroptosis.** (A, C) Indicated GPX4 (A) or GSTM1 (C) knockdown PC9-BrM3 cells were treated with different doses of carboplatin for 72 h and CCK-8 assays were performed to detect their viability. (n=3, **p<0.01) (B, D) Cell survival of indicated GPX4 (B) or GSTM1 (D) knockdown PC9-BrM3 cells with certain concentrations of carboplatin (0,20,60μM) treatment was evaluated by clonogenic assays. Pixel density quantification of clonogenic assays shown as histogram. (n=3, *p<0.05) Ctrl, control PC9-BrM3 cells; shNC, PC9-BrM3 cells transfected with negative control shRNA; shGPX4, PC9-BrM3 cells transfected with GPX4-targeted shRNA vector; shGSTM1, PC9-BrM3 cells transfected with GSTM1-targeted shRNA vector. (E-F) The cell viability of PC9 cells with indicated treatments for 72h. (n=3, **p<0.01) (G-J) PC9-BrM3 cells were treated with indicated treatments for 72h. The cell viability (G), levels of total iron(H), MDA (I) and lipid peroxidation (J) were assayed. (n=3, *p<0.05, **p<0.01) RSL3, (1S, 3R)-RSL3; Fer-1, ferrostatin-1; Cis, cisplatin; Carbo, carboplatin; TBH, tert-butyl hydroperoxide (200µM for 2 h) treatment was given as a positive control for BODIPY™ 581/591 C11 stain.


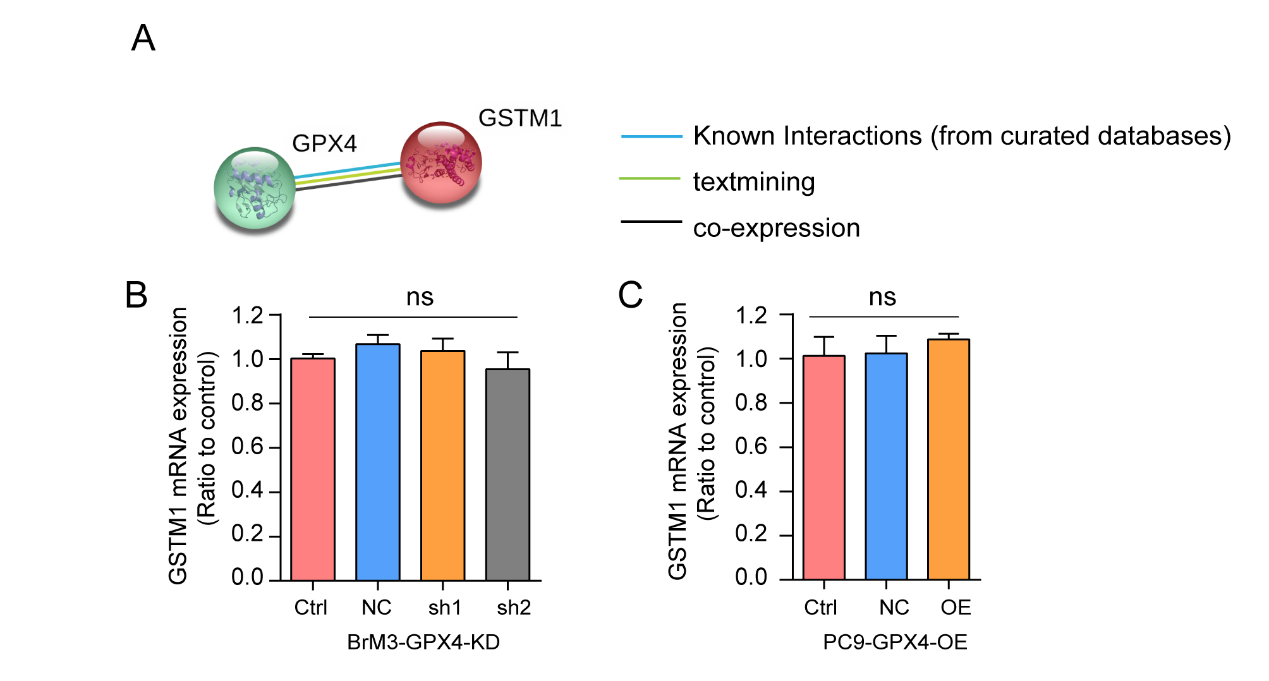
 **Figure S4. GPX4 regulated the level of GSTM1 by protein stabilization.** (A) Protein-protein interaction (PPI) of GPX4 and GSTM1 was predicted by STRING. (B) mRNA levels of GSTM1 in GPX4 knockdown PC9-BrM3 cells were determined by qPCR. KD, knock down; Ctrl, control PC9-BrM3 cells; shNC, PC9-BrM3 cells transfected with negative control shRNA; sh1, PC9-BrM3 cells transfected with GPX4-targeted shRNA vector 1; sh2, PC9-BrM3 cells transfected with GPX4-targeted shRNA vector 2. (n=3; ns, no statistic difference) (C) mRNA levels of GSTM1 in PC9 cells with GPX4 overexpression were determined by qPCR. Ctrl, control PC9 cells; NC: PC9 transfected with negative control plasmid; OE, PC9 transfected with GPX4 plasmid. (n=3; ns, no statistic difference)


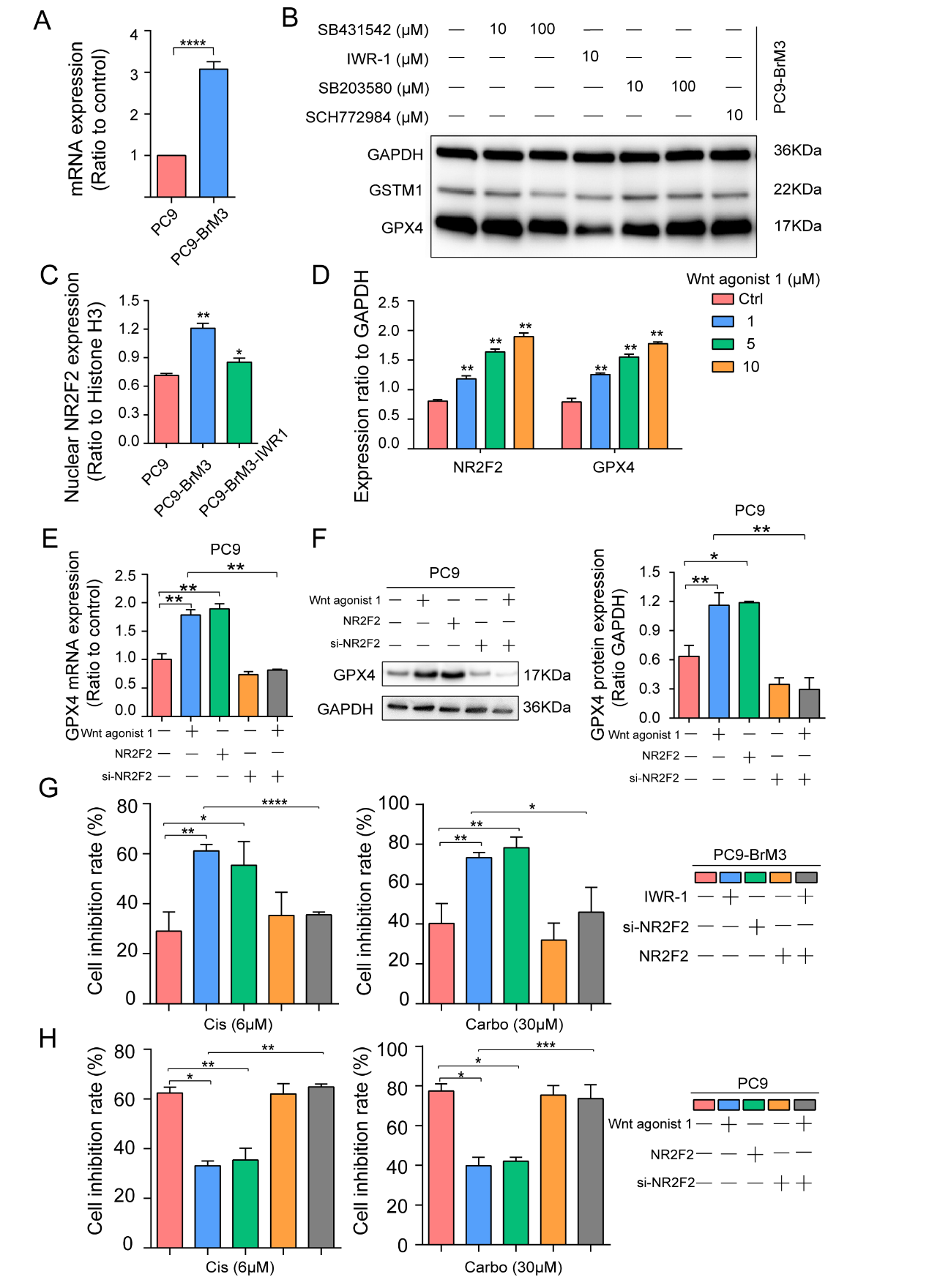


**Figure S5. Wnt/NR2f2 signaling is responsible for transcriptional upregulation of GPX4 in BM.** (A) mRNA levels of GPX4 in PC9 and PC9-BrM3 cells were determined by qPCR. (n=3, ****p<0.0001) (B) Representative western blot image showing the GPX4 and GSTM1 expression in PC9-BrM3 cells treated with different pathway inhibitors for 48h. (C) The bar graphs showing the nuclear NR2F2 expression in PC9, PC9-BrM3 cells, and PC9-BrM3 cells treated with Wnt inhibitor IWR-1-endo for 48h. Histone H3 acted as the internal reference protein for nuclear protein. (n=3, *p<0.05, **p<0.01, versus PC9 group) (D) The bar graphs showing the expressions of NR2F2 and GPX4 in PC9 cells treated with Wnt agonist 1 (1 μM, 5 μM, 10 μM) for 48h. (n=3, *p<0.05, **p<0.01, versus Ctrl group) (E-F) Results of qPCR(E) and Western-blot (F) showing the mRNA and protein expressions of GPX4 in PC9 cells after treatment with the Wnt agonist 1 (5 μM) with or without transfection with NR2F2 siRNA, or transfected with NR2F2 plasmid or NR2F2 siRNA alone for 48h. Representative images of each group are presented and the bar graphs are summarized results from 3 independent experiments. (n=3, *p<0.05, **p<0.01). (G-H) The cell inhibition rate of PC9-BrM3 cells (G) and PC9 cells (H) in indicated groups were treated with cisplatin or carboplatin for 72h. (n=3, *p<0.05, **p<0.01, ****p<0.0001) si-NR2F2, PC9-BrM3 or PC9 cells transfected with NR2F2 siRNA oligo; NR2F2, PC9-BrM3 or PC9 cells transfected with NR2F2 plasmid; Cis, cisplatin; Carbo, carboplatin.


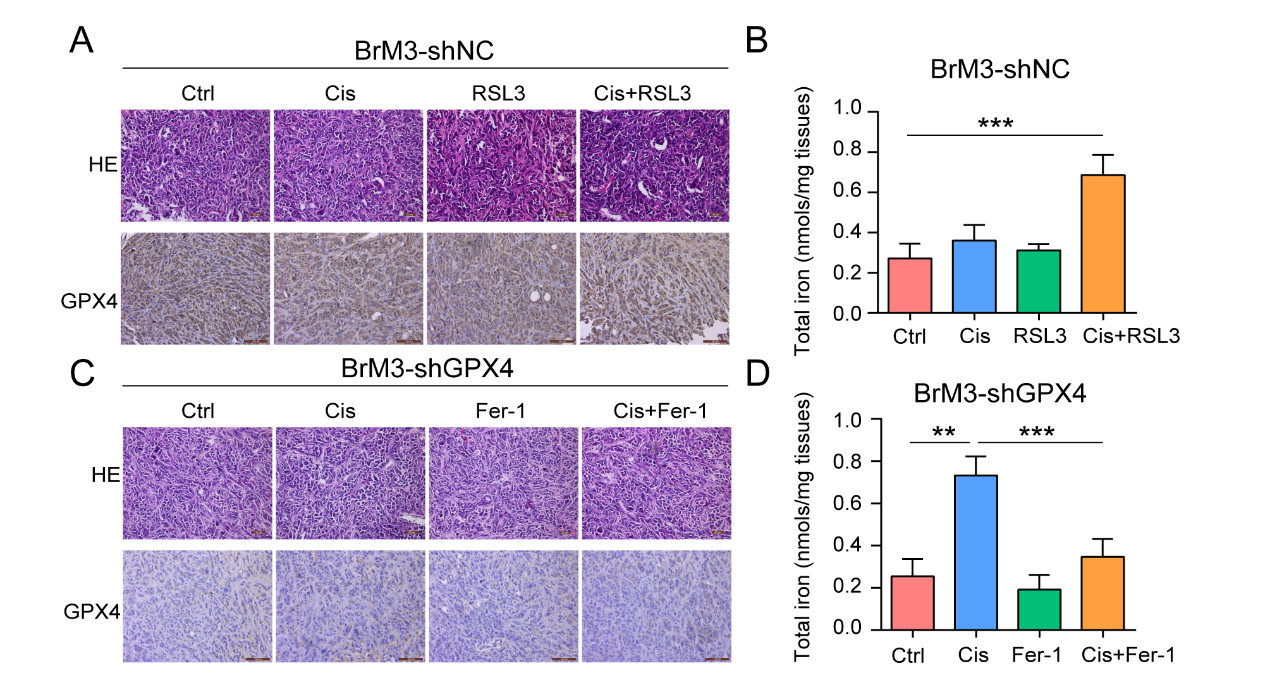


**Figure S6. GPX4 targeting inhibitor therapeutically enhances the anticancer activity of platinum *in vivo*.** (A,C) HE and IHC staining for obtained subcutaneous masses. (B,D) Total iron assessments for obtained subcutaneous masses. BrM3-shNC, PC9-BrM3 cells with negative control shRNA transfection; BrM3-shGPX4, PC9-BrM3 cells with GPX4 shRNA transfection; Ctrl, DMSO; Cis, cisplatin; Carbo, carboplatin; Fer-1, ferrostatin-1.

Table S1 Details of the metabolite features annotation and verification.

| Metabolites | Retention Time (minutes) | Retention Index | Similarity | Verification* |
| --- | --- | --- | --- | --- |
| Ethanolamine | 13.8 | 1263.1 | 832 | Yes |
| Succinate | 15.2 | 1312.5 | 890 | Yes |
| Glycerate | 15.6 | 1328.5 | 914 | Yes |
| Cysteine 1 | 19.1 | 1463.9 | 715 | Yes |
| Cysteine | 21.3 | 1551.6 | 911 | Yes |
| D-Ribose 5-Phosphate | 32.9 | 2103.9 | 805 | Yes |
| D-Ribulose 5-phosphate | 33.1 | 2115.6 | 783 | Yes |
| D-Fructose 6-Phosphate | 36.4 | 2301.8 | 711 | Yes |
| D-Glucose 6-phosphate 1 | 36.6 | 2313.0 | 847 | Yes |
| D-Glucose-6-phosphate 2 | 36.9 | 2330.8 | 767 | Yes |

* The features were verified by RT/RI using chemical standards
